# A Study of Antioxidant, Antihyperlipidemic, and Anti-Glycation Effects of Alkylsulfonic Acids with Quinobenzothiazinyl Substituents: In Vitro and In Silico Investigations

**DOI:** 10.3390/antiox14040464

**Published:** 2025-04-12

**Authors:** Kirthani Anamalay, Lee Qiao Er, Abbirami Balachandran, Patrick Nwabueze Okechukwu, Beata Morak-Młodawska, Merell P. Billacura, Charlie A. Lavilla, Anis Najwa Abdul Rani, Anand Gaurav, Adam Konefał, Małgorzata Jeleń

**Affiliations:** 1 Department of Biotechnology, Faculty of Applied Sciences, Ucsi University, No. 1 Jalan Menara Gading, UCSI Heights (Taman Connaught), Cheras, Kuala Lumpur 56000, Malaysia; kirthani1296@gmail.com (K.A.); 1002370495@ucsiuniversity.edu.my (L.Q.E.); 1001233455@ucsiuniversity.edu.my (A.B.); 2 Department of Pharmacology, Faculty of Pharmacy, Capital City University, No. 20 Yusuf Maitama Sule Road, Nasarawa GRA, Kano PMB 3409, Nigeria; 3 Department of Organic Chemistry, Faculty of Pharmaceutical Sciences, Medical University of Silesia, Jagiellońska, Str. 4, 41-200 Sosnowiec, Poland; bmlodawska@sum.edu.pl; 4 Department of Chemistry, College of Natural Sciences and Mathematics, Mindanao State University-Main Campus, Marawi City 9700, Philippines; merell.billacura@msumain.edu.ph; 5 Chemistry Department, College of Science & Mathematics, Mindanao State University-Iligan Institute of Technology, Iligan City 9200, Philippines; charliejr.lavilla@g.msuiit.edu.ph; 6 Faculty of Pharmaceutical Sciences, UCSI University, Cheras, Kuala Lumpur 56000, Malaysia; 1002265655@ucsiuniversity.edu.my (A.N.A.R.); anand.gaurav@ddn.upes.ac.in (A.G.); 7 Institute of Physics, University of Silesia in Katowice, 40-007 Katowice, Poland; adam.konefal@us.edu.pl

**Keywords:** antihyperlipidemic, quinobenzothiazine, BSA-MGO, BSA-GLU, ROS, cholesterol esterase, pancreatic lipase, phenothiazine

## Abstract

Hyperlipidemia, marked by high levels of fats in the blood, is a major risk factor for non-communicable diseases such as type 2 diabetes, cardiovascular diseases, and cancer. It has been linked to the action of reactive oxygen species and the formation of advanced glycation end products. Current treatments for hyperlipidemia, like orlistat, simvastatin, and atorvastatin, often present undesirable side effects, prompting the need for new therapeutic agents that are safer, more effective, cost-efficient, and have fewer side effects. In this context, new compounds, specifically propano- and butanosulfonic acids with 9-substituted quinobenzothiazinyl substituents, were synthesized through reactions with 9-substituted quinobenzothiazines and propane sultone or butane sultone. These novel quinobenzothiazine derivatives were verified using ^1^H NMR, ^13^C NMR, and HR-MS techniques. The research focused on assessing these compounds for their toxicity, ability to prevent glycation, antioxidant properties, and their potential to combat hyperlipidemia. Toxicity was evaluated on the 3T3 L1 fibroblast cell line using the MTT assay. The capacity to prevent glycation was tested with bovine serum albumin–methylglyoxal and bovine serum albumin–glucose systems. This study measured total reactive oxygen species in the 3T3 L1 cell line using 2′,7′-dichlorodihydrofluorescein diacetate staining, and antioxidant capacity was assessed through DPPH scavenging and metal ion chelation tests. The effectiveness against hyperlipidemia was determined by targeting cholesterol esterase and pancreatic lipase activities, with concentrations of the compounds **5** to **12** ranging from 0.0245 to 0.268 μM. Standard drugs such as orlistat, simvastatin, statins, and aminoguanidine were used as positive controls in various assays. Additionally, computational docking studies with AutoDock Vina were performed. The resulting findings indicated that the compounds were non-toxic to cells, effectively inhibited key enzymes related to hyperlipidemia, and showed significant antioxidant properties, including the prevention of advanced glycation end-product formation. Compounds **11** and **12** demonstrated the highest activity levels. These promising results highlight the potential of new quinobenzothiazine derivatives as lead compounds for the development of antihyperlipidemic drugs, although further research is necessary to confirm their efficacy and safety.

## 1. Introduction

Sulfur-containing compounds are prevalent in a broad spectrum of active biological, pharmaceutical, and natural molecules [1,2]. Organosulfur substances that have found use as drugs can be divided into 10 different categories with different structures and containing sulfur of different valencies, including sulfonamide, sulfone, sulfoxide, thioester, thioether, thiophene, thiazole, β-lactam, thiazepine/thiazine, and thiadiazole [3,4]. Almost 30% of drugs containing sulfur are estimated to be sulfonamides. These are synthetic substances derived from sulfanilamide (p-aminobenzenesulfonamide) that proved to be effective treatments for bacterial infections and diabetes until the 1940s. From sulfanilamide, more than 150 different chemically modified derivatives appeared on the market to achieve a more effective antibacterial effect, a wider spectrum of infected microorganisms, or a longer duration of action [5,6]. Sulfonamides also have a broad biological profile, as they are known to have, among others, antibacterial [7,8], hypoglycemic [9], diuretic [10,11], carbonic anhydrase [12], antifungal [13], anti-inflammatory [14], anticonvulsant [15], antioxidant [16], antitubercular [17], and anti-cancer properties [18,19,20]. These relationships are relatively inexpensive to produce and are still used in many countries in the world for the treatment of fungal diseases in combination with other drugs synergistically. Currently, sulfonamide drugs are enjoying renewed interest for the treatment of infections caused by bacteria resistant to other antibiotics [21,22]. Sulfonamide-containing compounds can be considered as bioisosteres of amides with better stability toward proteolytic hydrolysis in the plasma and gut. Similarly, acyl sulfonamides are widely used as carboxylic acid bioisosteres with improved pharmacological properties, such as plasma protein binding and permeability [23].

Organosulfur compounds that are also important from the point of view of medicinal chemistry are substances containing sulfonate groups. They constitute important pharmaceutical intermediates and are widely used in the synthesis of drugs, including sulfonamides. Substances containing sulfonate substituents are characterized by high polarity and often good solubility in water; the hydroxyl group in the sulfonic acid group may be a hydrogen-bond donor, while the oxygen atom can act as a hydrogen-bond receptor. This means that these compounds can bind to the molecular target, improving their binding affinity, and consequently, also improving their biological activity [24]. A wide spectrum of biological activities has been demonstrated for some substances containing a sulfonic acid substituent. This type of substance includes suramin, taurine, and its analogs. For suramin, originally used to treat *Trypanosoma brucei rhodesiense*, has also been shown to be active, among others, as an anti-insect, anti-inflammatory, and anti-cancer agent [25]. However, taurine has been patented for use in acute hepatitis, congestive heart failure, epilepsy, hypertension, and diabetes, among others. A number of taurine analogs with interesting pharmacological profiles have also been synthesized. Compounds of this type are characterized by, among others, protective properties for the nervous system and retina, as well as anti-alcoholic and anti-cancer properties. Taurine nitrourea is used clinically as an anti-cancer agent, and acamprosate is widely used in the treatment of alcoholics. Taurine analogs are also used against diabetes and help rebuild bone loss with stimulation of bone formation [26].

In the course of our previous research on phenothiazines modified with the quinoline ring, we obtained a number of different quinobenzothiazines and diquinothiazines with high biological potential. Selected N-substituted quinobenzothiazines show significant anti-cancer activity against dozens of cancer cells originating from leukemia, melanoma, non-small cell lungs, colon, CNS, ovary, kidney, prostate, breast, and skin cancer. These compounds also show promising antioxidant effects, inhibitory activity effects on mitogen-induced proliferation of human peripheral blood mononuclear cells, tumor necrosis factor alpha (TNFα) production in human whole blood cultures, and butyrylcholinesterase. Some quinobenzothiazines exerted suppressive effects in in vivo models of delayed-type hypersensitivity to ovalbumin and skin reaction to carrageenan, contact sensitivity to oxazolone, and psoriasis in mice; they showed inhibitory effects of IFNβ expression and downstream IFNβ-dependent genes and proteins involved in the pathogenesis of autoimmune diseases [27,28,29,30,31].

Abnormally elevated lipid or lipoprotein levels in the blood are defined as hyperlipidemia or hyperlipoproteinemia. Hyperlipidemia is the most prevalent form of dyslipidemia, characterized by elevated lipid levels. Hyperlipidemia includes an array of diseases marked by elevated concentrations of lipids, including triglycerides, phospholipids, cholesterol, and cholesteryl esters in the blood.

Hyperlipidemia is classified into two categories: primary (familial), resulting from genetic defects, and secondary (acquired), due to underlying disorders. Hyperlipidemia can be further categorized according to the specific forms of raised lipids, including hypercholesterolemia (elevated cholesterol) and hypertriglyceridemia (elevated triglyceride levels). Secondary hyperlipidemia, frequently resembling familial hyperlipidemia, may produce similar outcomes. Both primary and secondary hyperlipidemia can increase the risk of early atherosclerosis. Pancreatitis and other problems associated with chylomicronemia syndrome may arise in instances of severe hypertriglyceridemia.

Hyperlipidemia is becoming increasingly prevalent in many countries due to evolving food choices and a sedentary lifestyle. The transition to a diet that is characterized by a high intake of processed foods, trans fats, and saturated fats is a consequence of fast economic expansion and urbanization in several countries. The prevalence of hyperlipidemia has risen in nations like China, India, and Japan because of dietary modifications and reduced physical activity. A notable health hazard in Malaysia is hyperlipidemia. The Malaysian National Health and Morbidity Survey 2019 indicated that hypercholesterolemia, a form of hyperlipidemia, was present in around 38.1% of persons aged 18 and older with elevated cholesterol levels. Multiple factors contribute to its prevalence, including the significant consumption of trans fats and saturated fats in traditional Malaysian cuisine, a sedentary lifestyle, and rising obesity rates. Obesity is marked by insulin resistance, a precursor to type 2 diabetes mellitus that leads to hyperlipidemia. Obese people with underlying insulin resistance have high insulin concentrations in the blood [32]. Their adipocytes (fat cells) both express abnormal genes and their products, which promote lipolysis, as is shown by a significant increase in hormone-sensitive lipase messenger ribonucleic acid fluxes in the body fats compared to lean controls; this empowers them to release even more fatty acids into the bloodstream [33].

Since 1975, the worldwide incidence of obesity has roughly tripled, impacting several nations, especially those with low to average earnings. A countrywide study in Malaysia performed in 2019 indicated an escalating trend in obesity prevalence, which increased from 15.1% in 2011 to 17.7% in 2015 and ultimately to 19.9% in 2019 [34]. The incidence rate of obesity in Malaysia keeps rising; experts cannot help but become increasingly concerned about what might happen to the overall health picture. Factors in obesity include changes to dietary routines, inactive lifestyles, and the urbanization of society [35]. This article examines the present state of weight problems in Malaysia, including obesity statistics, Body Mass Index (BMI) trends, and potential consequences for public health research and public policy. Understanding the relationship between obesity and adult BMI categories with related chronic conditions is essential in devising appropriate interventions to address this growing health crisis [34].

In these studies, we focused on modifying the structure of quinobenzothiazines with substituents containing a sulfonic acid fragment and on assessing them for toxicity, anti-glycation capacity, antioxidant properties, and their potential to combat hyperlipidemia.

## 2. Materials and Methods

### 2.1. Chemistry

#### 2.1.1. General Methods

Melting points were determined in open capillary tubes on a Boetius melting point apparatus and were uncorrected. The standard NMR spectra were recorded on Bruker Avance spectrometers (Bruker, Billerica, MA, USA) (^1^H at 600 MHz, ^13^C at 150 MHz) in DMSO-d_6_. The HRMS spectra (EI-electroimpact ionization) were run on a Brucker Impact II (Bruker, Billerica, MA, USA). ^1^HNMR, ^13^CNMR, and HRMS spectra are included in Supplementary Materials.

#### 2.1.2. General Procedure for the Synthesis of 3-(Quino [3,2-b]benzo[1,4]thiazin-6-yl) Propane-1-sulfonic Acids **5**–**8**

To a solution of 9-substituted 6*H*-quinobenzothiazine **1**, **2**, **3,** or **4** (0.5 mmol) in 5 mL dry DMF, NaH (0.06 g, 2.5 mmol 60% NaH in mineral oil was washed out with hexane) was added. The reaction mixture was stirred at room temperature for 1 h, 1,3-propanesultone (0.12 g, 1 mmol) was added, and the stirring was continued for 24 h. The reaction mixture was poured into water (25 mL) and acidified to a pH of about 5 with 5% hydrochloric acid. The resulting solid was filtered off and washed with water to give compounds **5**–**8**:

3-(quino[3,2-b]benzo[1,4]thiazin-6-yl)propane-1-sulfonic acid (**5**):

Yield: 65%. M.p.: 183–184 °C. ^1^H NMR (DMSO-d_6_) δ: 2.09–2.12 (m, 2H, CH_2_), 2.63 (t, 2H, CH_2_, *J* = 7.2 Hz), 4.33 (t, 2H, CH_2_, *J* = 7.2 Hz), 7.00 (t, 1H, H-9, *J* = 7.2 Hz), 7.18–7.19 (m, 1H, H-7), 7.24 (t, 1H, H-2, *J* = 7.2 Hz), 7.27 (d, 1H, H-10, *J* = 7.2 Hz), 7.33 (t, 1H, H-3, *J* = 7.8), 7.56, (t, 1H, H-8, *J* = 7.2 Hz), 7.67, (d, 1H, H-1, *J* = 7.2 Hz), 7.70 (d, 1H, H-4, *J* = 7.2 Hz), 7.98 (s, 1H, H-12). ^13^C NMR (75 MHz, DMSO-d_6_) δ: 22.65, 40.51, 44.70, 49.16, 117.10, 118.19, 119.58, 123.68, 124.92, 125.93, 126.56, 126.96, 127.14, 128.58, 130.08, 132.79, 140.70, 144.60, 151.91. HR MS (ESI) calcd for C_18_H_17_N_2_O_3_S_2_ [M + H]^+^: 373.0681, found: 373.0672.

3-(9-fluoroquino[3,2-b]benzo[1,4]thiazin-6-yl)propane-1-sulfonic acid (**6**):

Yield: 56%. M.p.: 180–181 °C. ^1^H NMR (DMSO-d_6_) δ: 2.08–2.10 (m, 2H, CH_2_), 2.65 (t, 2H, CH_2_, *J* = 7.2 Hz), 4.31 (t, 2H, CH_2_, *J* = 7.2 Hz), 7.07–7.09 (m, 1H, H-9), 7.17–7.19 (m, 1H, H-7), 7.26–7.29 (m, 1H, H-10), 7.33 (t, 1H, H-2, *J* = 7.8 Hz), 7.56 (t, 1H, H-3, *J* = 7.8), 7.71 (d, 1H, H-1, *J* = 7.2 Hz), 7.70, (d, 1H, H-4, *J* = 8.2 Hz), 8.00 (s, 1H, H-12). ^13^C NMR (75 MHz, DMSO-d_6_) δ: 22.52, 40.51, 44.86, 114.05 (*J* = 25.5 Hz), 114.81 (*J* = 21 Hz), 117.39, 118.25 (*J* = 9 Hz), 121.86 (*J* = 9 Hz), 124.96, 125.81, 127.00 (*J* = 9.0 Hz), 130.24, 133.12, 137.26, 144.86, 150.95, 151.80, 158.46 (*J* = 241.5 Hz). HR MS (ESI) calcd for C_18_H_16_FN_2_O_3_S_2_ [M + H]^+^: 391.0586, found: 391.0591.

3-(9-chloro[3,2-b]benzo[1,4]thiazin-6-yl)propane-1-sulfonic acid (**7**):

Yield: 70%. M.p.: 192–193 °C. ^1^H NMR (DMSO-d_6_) δ: 2.05–2.09 (m, 2H, CH_2_), 2.60 (t, 2H, CH_2_, *J* = 7.2 Hz), 4.30 (t, 2H, CH_2_, *J* = 7.2 Hz), 7.24–7.25 (m, 2H, H-7, H-10), 7.31–7.32 (m, 2H, H-2, H-8), 7.54–7.57 (m, 1H, H-3), 7.65- 7.68 (m, 2H, H-1, H-4), 7.98 (s, 1H, H-12). ^13^C NMR (75 MHz, DMSO-d_6_) δ: 22.65, 44.57, 49.22, 117.22, 118.21, 121.90, 124.91, 126.06, 126.30, 127.00, 127.09, 127.23, 128.04, 130.06, 132.85, 140.08, 145.35, 151.65. HR MS (ESI) calcd for C_18_H_16_ClN_2_O_3_S_2_ [M + H]^+^: 407.0291, found: 407.0283.

3-(9-methylthio[3,2-b]benzo[1,4]thiazin-6-yl)propane-1-sulfonic acid (**8**):

Yield: 62%. M.p.: 197–198 °C. ^1^H NMR (DMSO-d_6_) δ: 2.07–2.09 (m, 2H, CH_2_), 2.46 (s, 3H, CH_3_), 2.63 (t, 2H, CH_2_, *J*= 7.2 Hz), 4.30 (t, 2H, CH_2_, *J* = 7.2 Hz), 7.10–7.13 (m, 2H, H-7, H-10), 7.20 (d, 1H, H-8, *J* = 8.4 Hz), 7.31 (m, 1H, H-2), 7.55 (m, 1H, H-3), 7.66- 7.68 (m, 2H, H-1, H-4), 7.97 (s, 1H, H-12). ^13^C NMR (75 MHz, DMSO-d_6_) δ: 22.64, 34.49, 44.53, 49.15, 117.51, 117.81, 120.82, 124.85, 124.90, 125.88, 126.69, 126.84, 126.99, 130.08, 132.56, 132.80, 138.24, 144.83, 151.72. HR MS (ESI) calcd for C_19_H_19_N_2_O_3_S_2_ [M + H]^+^: 419.0558, found: 419.0557.

#### 2.1.3. General Procedure for the Synthesis of 4-(Quino[3,2-b]benzo[1,4]thiazin-6-yl) Butane-1-sulfonic Acids **9**–**12**

To a solution of 9-substituted 6*H*-quinobenzothiazine **1**, **2**, **3,** or **4** (0.5 mmol) in 5 mL dry DMF, NaH (0.06 g, 2.5 mmol 60% NaH in mineral oil was washed out with hexane) was added. The reaction mixture was stirred at room temperature for 1 h, 1,4-propanesultone (0.14 g, 1 mmol) was added, and the stirring was continued for 24 h. The reaction mixture was poured into water (25 mL) and acidified to a pH of about 5 with 5% hydrochloric acid. The resulting solid was filtered off and washed with water to give compounds **9**–**12**:

4-(quino[3,2-b]benzo[1,4]thiazin-6-yl)butane-1-sulfonic acid (**9**):

Yield: 68%. M.p.: 167–168 °C. ^1^H NMR (DMSO-d_6_) δ: 1.76–7.77 (m, 2H, CH_2_), 1.83–1.84 (m, 2H, CH_2_), 2.56 (t, 2H, CH_2_, *J* = 7.8 Hz), 4.25 (m, 2H, CH_2_), 6.98 (m, 1H, H-9), 7.14 (d, 1H, H-7, *J* = 7.8 Hz), 7.18 (m, 1H, H-2), 7.23 (t, 1H, H-10, *J* = 7.2 Hz), 7.31 (t, 1H, H-3, *J* = 7.8), 7.54, (m, 1H, H-8), 7.66–7.68 (m, 2H, H-1, H-4), 7.96 (s, 1H, H-12). ^13^C NMR (75 MHz, DMSO-d_6_) δ: 23.12, 25.55, 44.91, 51.47, 116.87, 118.10, 119.68, 123.41, 124.75, 126.05, 126.93, 127.07, 127.18, 128.57, 129.93, 132.59, 140.94, 145.24, 152.14. HR MS (ESI) calcd for C_19_H_19_N_2_O_3_S_2_ [M + H]^+^: 387.0837, found: 387.0848.

4-(9-fluoroquino[3,2-b]benzo[1,4]thiazin-6-yl)butane-1-sulfonic acid (**10**):

Yield: 60%. M.p.: 188–189 °C. ^1^H NMR (DMSO-d_6_) δ: 1.76–1.77 (m, 2H, CH_2_), 1.82–1.83 (m, 2H, CH_2_), 2.60 (t, 2H, CH_2_, *J* = 7.2 Hz), 4.20 (m, 2H, CH_2_), 7.06–7.08 (m, 1H, H-7), 7.13–7.18 (m, 2H, H-8, H-10), 7.31 (m, 1H, H-2), 7.53–7.56 (m, 1H, H-3, *J* = 7.8), 7.66–7.68 (m, 2H, H-1, H-4), 7.97 (s, 1H, H-12). ^13^C NMR (75 MHz, DMSO-d_6_) δ: 22.98, 25.39, 45.08, 51.39, 114.08 (*J* = 25.5 Hz), 114.80 (*J* = 21 Hz), 117.32, 118.02 (*J* = 9 Hz), 121.95 (*J* = 36.0 Hz), 124.79, 125.93, 127.00 (*J* = 9 Hz), 130.09, 132.95, 137.49, 145.22, 148.99, 152.05, 156.86 (*J* = 240.0 Hz). HR MS (ESI) calcd for C_19_H_18_FN_2_O_3_S_2_ [M + H]^+^: 405.0743, found: 405.0748.

4-(9-chloro[3,2-b]benzo[1,4]thiazin-6-yl)butane-1-sulfonic acid (**11**):

Yield: 76%. M.p.: 165–166 °C. ^1^H NMR (DMSO-d_6_) δ: 1.75–1.76 (m, 2H, CH_2_), 1.80–1.81 (m, 2H, CH_2_), 2.55 (t, 2H, CH_2_, *J* = 7.8 Hz), 4.20 (m, 2H, CH_2_), 7.13 (d, 1H, H-7, *J* = 9 Hz), 7.25 (m, 1H, H-9), 7.31–7.34 (m, 2H, H-2, H-8), 7.56, (m, 1H, H-3), 7.66–7.68 (m, 2H, H-1, H-4), 7.98 (s, 1H, H-12). ^13^C NMR (75 MHz, DMSO-d_6_) δ: 23.08, 25.41, 44.92, 51.46, 117.21, 118.11, 122.00, 124.91, 126.07, 126.34, 127.00, 127.05, 127.26, 128.10, 130.09, 132.90, 140.05, 145.39, 151.74. HR MS (ESI) calcd for C_19_H_18_ClN_2_O_3_S_2_ [M + H]^+^: 421.0447, found: 421.0455.

4-(9-methylthio[3,2-b]benzo[1,4]thiazin-6-yl)butane-1-sulfonic acid (**12**):

Yield: 64%. M.p.: 172–173 °C. ^1^H NMR (DMSO-d_6_) δ: 1.74–1.76 (m, 2H, CH_2_), 1.81–1.83 (m, 2H, CH_2_), 2.21 (s, 3H, CH_3_), 2.56 (t, 2H, CH_2_, *J* = 6.6 Hz), 4.20 (m, 2H, CH_2_), 6.99 (m, 1H, H-7), 7.03 (m, 1H, H-10), 7.05 (d, 1H, H-8, *J* = 8.4 Hz), 7.30 (t, 1H, H-2, *J* = 7.2 Hz), 7.54 (m, 1H, H-3), 7.64–7.67 (m, 2H, H-1, H-4), 7.94 (s, 1H, H-12). ^13^C NMR (75 MHz, DMSO-d_6_) δ: 23.07, 25.57, 34.50, 44.81, 51.45, 116.75, 118.18, 119.48, 124.62, 125.90, 126.85, 126.91, 127.44, 129.01, 129.90, 132.52, 132.68, 138.34, 145.13, 151.14. HR MS (ESI) calcd for C_20_H_21_N_2_O_3_S_2_ [M + H]^+^: 433.0714, found: 433.0731.

### 2.2. MTT Cell Viability Assay

The viability of 3T3-L1 cells purchased from ATTC (CL-173) was assessed using an MTT assay. The assay was conducted according to the methodology outlined by Vajrabhaya and Korsuwannawong [36]. Cells were initially seeded at a density of 10^−4^ cells per well in a 96-well plate and incubated for 24 h at 37 °C in a 5% CO_2_ environment. Following incubation, the cells were subjected to different concentrations and incubated for a duration of 24 h. Each well, containing 100 μL, was filled with MTT stock solution at a concentration of 0.5 mg/mL to assess cell viability. The plate underwent incubation for a duration of three hours. Living cells transform the yellow tetrazolium salt MTT into a purple formazan product. Following incubation, the MTT solution was removed, and 100 μL of dimethyl sulfoxide (DMSO) was added to each well. The formazan crystals were dissolved by gently shaking the plate and allowing it to remain in the dark for fifteen minutes. The absorbance of the formazan product was measured at a wavelength of 540 nm using a microplate reader. The absorbance value is directly correlated with the number of viable cells in each well. This assay quantitatively assesses cell viability and can evaluate cytotoxicity or the potential effects of the synthetic compound on 3T3-L1 cells.

### 2.3. Intracellular ROS Assay

#### 2.3.1. 3T3-L1 Differentiation of Preadipocytes into Adipocytes

Differentiation of 3T3-L1 cells was performed within one week through culturing in MDI medium, which consisted of 0.5 mM 3-isobutyl-1-methylxanthine, 1 μM dexamethasone, and 1 μg/mL insulin, for 48 h in 10% FBS-DMEM. Subsequently, the cells were transferred to fresh 10% FBS and 90% DMEM supplemented with 1 μg/mL insulin. MDI addition marks day 0 of the differentiation protocol; following this treatment, 70–80% of cells exhibited a significant increase in triglyceride content within 7 to 14 days.

#### 2.3.2. Assessment of Intracellular ROS Production

On day 8 of differentiation, intracellular ROS levels in 3T3 L1 cells were assessed using the oxidation-sensitive fluorogenic probe 2′,7′-dichlorodihydrofluorescein-diacetate (DCFH-DA), following a method established by Forbes-Hernández et al., with minor modification [37]. Post-differentiation, the cells were treated with the respective doses of the compounds for a period of 24 h. Following this, the cells underwent incubation with DCFH-DA (25 μM) at 37 °C for a duration of 30 min in the absence of light. Consequently, the cells were stimulated with 1 mM AAPH as a ROS initiator for a duration of 1 h. The density of light was measured using a spectrofluorometer with an excitation wavelength of 480 nm and an emission wavelength of 530 nm.

### 2.4. DPPH Radical Scavenging Assay

The DPPH (2,2-diphenyl-1-picrylhydrazyl) radical scavenging assay was conducted to evaluate the antioxidant activity of compounds **5**–**12** following the method outlined by Chong et al. [38] with minor modifications. A 1 mL sample with a concentration range of 0.0245 to 0.268 μM was introduced to a 1 mL methanol solution of DPPH (0.05 M) and vortexed. Following a 20-min incubation at 25 °C, absorbance was assessed at 527 nm using a UV-vis spectrophotometer, with blank as reference. Ascorbic acid served as a positive control. The negative control consists of a setup without samples (ascorbic acid and synthetic compounds). The percentage of DPPH free radical inhibition was determined using Equation (1) provided below.

% of DPPH radical scavenging activity was calculated using the following equation:

(1) $$AA\%=100-[\frac{\left(Abs\;sample-Abs\;blank\right)100}{Abs\;control}]$$

Equation (1) where:
Abs control = absorbance of the control.
Abs sample = absorbance of the sample.

### 2.5. Chelating Capacity

Chelating capacity assay is conducted following the method outlined by Chong et al. [38] with minor modifications. Samples of 0.1 mL with concentrations ranging from 0.0245 to 0.268 μM were combined with 0.5 mL of a 2 mM solution and 0.2 mL of a 5 mM ferrozine solution. Then, a 10-min incubation at room temperature, absorbance was recorded at 562 nm using a blank solution in a 96-well plate reader. EDTA served as a positive control. The negative control consists of a sample devoid of any substances, including EDTA and synthetic compounds. The percentage of ferrous chelation was calculated using the Equation (1).

### 2.6. In Vitro Antihyperlipidaemia Assays

#### 2.6.1. In Vitro Pancreatic Lipase Assay

The pancreatic lipase inhibition assay is conducted following the method outlined by Ajayi et al. (2022) with minor modifications [39]. The enzyme source utilized was porcine pancreas powder at a concentration of 0.1 mg·mL^−1^ in Tris–HCl buffer, pH 8.0. In a 96-well plate, 50 μL of phosphate–buffer solution (pH 7.0), 50 μL of pNPB (500 μM), and 50 μL of test samples **5**–**12** with concentrations ranging from 0.0245 to 0.268 μM were pipetted and mixed thoroughly. Fifty microliters of pancreatic enzyme were added to the resultant mixture and incubated at room temperature for 30 min. The resulting yellow p-nitrophenol was measured spectrophotometrically at 405 nm. Control was conducted concurrently without the addition of a test sample. Orlistat served as a positive control. PL inhibition was determined using the following formula: PL inhibition (%) = [(Activity of control − activity of test)/activity of control] × 100. The IC_50_ is determined utilizing GraphPad 10 software (GraphPad Software, LLC, San Diego, CA, USA).

#### 2.6.2. In Vitro Cholesterol Esterase (CEase) Assay

A modified version of the method outlined by Gururaja et al. (2015) was utilized to assess the in vitro inhibitory activity of cholesterol esterase [40]. In a 96-well plate, 50 μL of phosphate–buffer solution (pH 7.0), 50 μL of pNPB (500 μM), and 50 μL of test samples **5**–**12** with concentrations ranging from 0.0245 to 0.268 μM were pipetted and mixed thoroughly. Fifty microliters of cholesterol esterase were added to the resultant mixture and incubated at room temperature for five minutes. The resulting yellow p-nitrophenol was measured spectrophotometrically at 405 nm. Control was conducted concurrently without the addition of a test sample. Simvastatin served as a positive control. The calculation for CEase inhibition is as follows: CEase inhibition (%) = [(Activity of control − Activity of test)/Activity of control] × 100. The IC_50_ is determined utilizing GraphPad software.

### 2.7. In Vitro Antiglycation Assays

#### 2.7.1. BSA-Methylglyoxal Inhibition Assay

The assay for methylglyoxal in bovine serum albumin (BSA) was conducted following the methodology outlined by Mridula et al. [41]. A reaction mixture totaling 3 mL comprised 1 mL of bovine serum albumin (BSA), 1 mL of methylglyoxal, and 1 mL of the aminoguanidine and **5**–**12** at various concentrations ranging from 0.0245 to 0.268 μM. The reaction mixture was thoroughly mixed and incubated for 5 min. Following incubation, 0.5 mL of sodium azide was added to each tube. The tubes were securely sealed and incubated in the dark at 37 °C for a duration of seven days. Aminoguanidine served as a positive control inhibitor for protein glycation. Following a seven-day incubation period, the samples were analyzed with the Omega microplate reader (BMG LABTECH, Ortenberg, Germany), measuring fluorescence intensity at an excitation wavelength of 370 nm and an emission wavelength of 420 nm. The experiment was conducted in triplicate.

#### 2.7.2. BSA-Glucose Inhibition Assay

The BSA-glucose assay is conducted according to the methodology outlined by Mridula et al. [41]. The glucose assay utilizing bovine serum albumin (BSA) was conducted with a 3 mL reaction mixture comprising 1 mL of BSA, 1 mL of glucose, and 1 mL of test compounds **5**–**12** with varying concentrations from 0.0245 to 0.268 μM, all prepared in a 0.1 M sodium phosphate buffer at pH 7.4. The reaction mixture was thoroughly mixed and incubated for 5 min. Following incubation, 0.5 mL of sodium azide was added to each tube. The tubes underwent incubation at 37 °C in the absence of light for a duration of seven days. Aminoguanidine is used as a positive control. Following a seven-day incubation period, the samples were analyzed with the Omega microplate reader (BMG LABTECH, Ortenberg, Germany), measuring fluorescence intensity at an excitation wavelength of 370 nm and an emission wavelength of 440 nm. The experiment was conducted in triplicate.

## 3. Results and Discussion

### 3.1. Synthesis of New Quinobenzothiazines Derivatives **5**–**12**

**Scheme 1:**
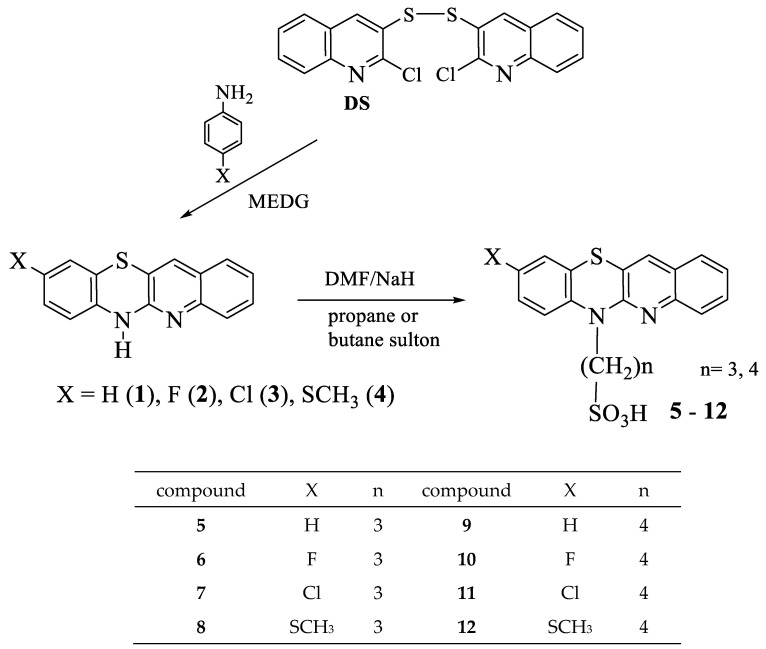
Synthesis of alkylsulfonic acids with quinobenzothiazinyl substituents **5**–**12**.

The corresponding quinobenzothiazines **1**–**4** (Scheme 1) were used to synthesize eight new propane- and butanesulfonic acids with quinobenzothiazinic, 9-fluoro-, 9-chloro-, and 9-methylthioquinobenzothiazinic substituents **5**–**12**. The quinobenzothiazines **1**–**4** were obtained by annulation reactions of 2,2′-dichloro-3,3′-diquinolyl disulfide with the appropriate *p*-substituted anilines according to previously developed and described procedures [42]. The title propane- and butanesulfonic acids were obtained in good yields by reactions of quinobenzothiazines **1**–**4** with propanesultone or butanesultone, respectively, according to the procedures described in Section 2.1. The structures of the new derivatives were confirmed by ^1^H NMR, ^13^C NMR, and HR MS analyses.

### 3.2. Biological Evaluation

#### 3.2.1. MTT Assy

The MTT assay shows the percentage inhibition of all treatment groups is lower at higher concentrations. Untreated cells were considered the control group, and orlistat cells were used as positive controls.

**Figure 1:**
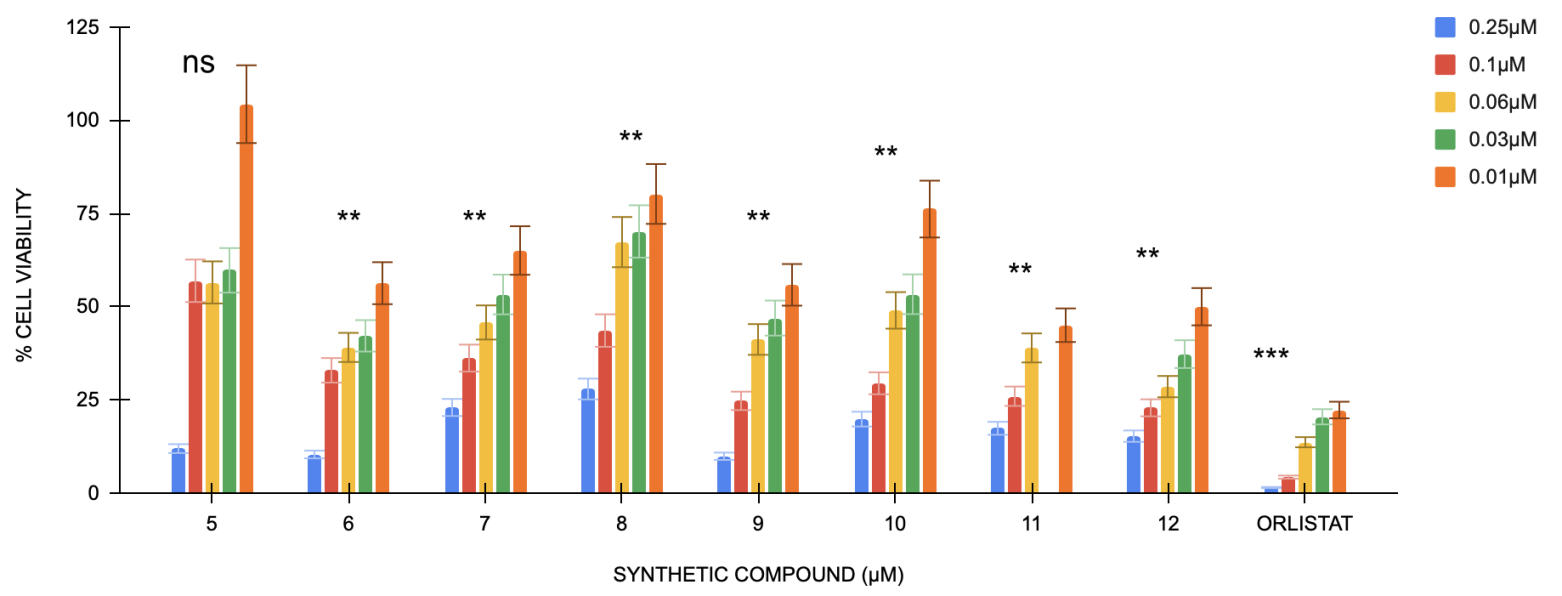
Cell viability percentages of compounds **5** to **12** against orlistat (*n* = 3). One-way ANOVA coupled with Dunnett’s multiple comparison test is represented by ** *p* < 0.01 and *** *p* < 0.001 against orlistat at their respective concentrations.

The absorbance values generally increase as the treatment concentration decreases (0.06 μM, 0.03 μM, and 0.01 μM) (Figure 1). This trend is observed across all compounds, indicating that lower concentrations are less detrimental to cell viability. The absorbance values for compound **5** show the highest at the lowest concentration (0.01 μM), indicating improved cell survival and cell proliferation at lower levels. Orlistat has a *p*-value < 0.001 compared with the control group, whereas compounds **9** and **7** have a *p*-value < 0.05, compounds **6**–**12** show a *p*-value < 0.01; however, compound **5** shows non-significance.

#### 3.2.2. Intracellular ROS Activity

The percentage of ROS generation in differentiated 3T3-L1 preadipocytes was conducted on various concentrations of compound **5**–**12** (Table 1). A dose-dependent decrease in ROS generation was observed for all eight compounds, with the highest ROS production occurring at lower concentrations and showing a decreasing percentage as concentration increases. Compound **5** shows the highest percentage inhibition at 0.02 μM (208.69 ± 13.07%) and is significantly reduced at higher concentrations. Moreover, compound **6** shows a reduction in ROS generation from 37.15 ± 9.37% at 0.2 μM to 63.73 ± 1.04% at 0.25 μM. Compound **7** shows a similar pattern, with ROS generation from 143.18 ± 10.65% at 0.02 μM to 14.96 ± 1.25% at 0.25 μM, and significant differences were noticed between the concentrations. Compound **8** also shows a similar pattern, with ROS generation at 0.02 μM (149.62 ± 6.71%) and a decrease to 50.89 ± 2.85% at 0.25 μM, showing a significant reduction in ROS generation at higher concentrations. For the compound **9**, ROS generation was 169.62 ± 13.81% at 0.02 μM and reduced to 26.38 ± 1.73% at 0.25 μM. Thus, it shows a significant difference between the concentrations. Compound **10** showed a dose-dependent reduction, ROS generation decreasing from 103.85 ± 1.17% at 0.02 μM to 47.16 ± 3.71% at 0.25 μM, and significant differences were observed. For compound **11**, ROS generation decreases from 104.90 ± 5.3% at 0.02 μM to 57.18 ± 1.66% at 0.25 μM, with significant differences between the concentrations. Lastly, compound **12** exhibited a slight reduction in ROS generation from 119.82 ± 5.12% at 0.02 μM to 60.12 ± 1.74% at 0.25 μM, showing a dose-dependent decrease in ROS generation with significant differences between concentrations. In conclusion, all compounds exhibited a dose-dependent reduction in ROS generation, with the highest ROS levels observed at the lowest concentrations.

#### 3.2.3. DPPH Radical Scavenging Assay

**Figure 2:**
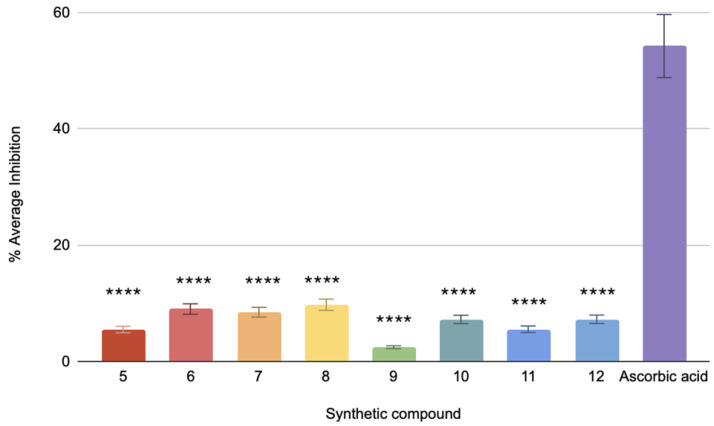
The % average inhibition of compound **5**–**12**. The results were demonstrated in triplicate (*n* = 3) analysis using one-way ANOVA. Different letters denote significant differences. **** *p* < 0.0001.

The DPPH assay for the antioxidant capacity of the compounds shows ascorbic acid at 54.24% mean inhibition, indicating a highly active antioxidant. Compounds **5**–**12** show inhibition rates of 5.49%, 3.45%, 8.47%, 7.36%, 2.45%, 6.21%, 8.47%, and 5.53%, respectively (Figure 2). Compounds **7** and **9** have a very high statistical significance compared to ascorbic acid, given that they contain a *p*-value of less than 0.0001, while the others have *p*-values of less than 0.001. Hence, these values are significantly far from ascorbic acid but less than compound 9. The relatively low percentage inhibition values obtained indicate that the above compounds exhibit lower antioxidant activity than ascorbic acid. Such a small inhibition will be affected by determining the IC_50_ values, or the required amount for 50% inhibition, and, hence, will not be suitable to perform within the tested range [43].

The DPPH test is a widely utilized method for assessing antioxidant activity by quantifying a compound’s ability to neutralize free radicals. The DPPH free radical frequently assesses the capacity of the compounds to function as free-radical scavengers and hydrogen donors, offering a quick, straightforward, cost-effective approach to evaluating antioxidant properties. The DPPH assay relies on the reduction in DPPH, a stabilized free radical. DPPH is a dark-hued crystalline molecule consisting of stable free-radical particles [44]. It is an acknowledged radical and a commonly employed antioxidant test. The DPPH radical initially exhibits a dark purple coloration in solution; however, following reduction and conversion to DPPH-H, it turns colorless or light yellow. Despite their promising structural properties, several variables linked to their structure and possible interactions might explain why the four compounds lack DPPH radical scavenging ability. The chemicals, which are usually linked to DPPH scavenging, first lack easily accessible hydroxyl (-OH), amine (-NH_2_), or thiol (-SH) groups [45]. Although they have a quinobenzothiazine core that can give hydrogen, their inability to contain easily accessible groups could impede their capacity to contribute a hydrogen atom to the DPPH radical directly. Ultimately, the sulfonic acid group and the quinobenzothiazine core might form intramolecular hydrogen bonds or participate in other interactions. These interactions could hinder the compounds’ capacity to interact with the DPPH radical by limiting the availability of possible hydrogen-donating sites or modifying their electrostatic properties [46]. Overall, despite the interesting structure of the compounds, their potential for DPPH radical scavenging was very low in comparison to ascorbic acid, probably because of the lack of accessible, functional groups; the sterically hindered environment; and electron-withdrawing effects. These features limit their capability to act as good hydrogen donors, hence the low antioxidant activity and inability to calculate their exact IC_50_ value.

#### 3.2.4. Chelating Capacity

Ion chelating inhibits catalysis of free radical generation via Fenton reactions, thereby decreasing oxidative stress. Chelating substances improve the antioxidant effectiveness of phenolic compounds by stabilizing the phenoxyl radical produced during free radical scavenging [29]. Because EDTA is an effective chelation agent, the results show that it exhibits increasing inhibitory action with increased concentration. Although possessing favorable structural characteristics, the compounds **5**–**12** do not exhibit chelating activity, which could be because they lack chelating qualities. This can be ascribed to a combination of variables mostly associated with their inherent limits in structure and the specific circumstances of the assay used. Chelation is a chemical process that involves the binding of metal ions. This binding is facilitated by the presence of certain functional groups that serve as highly effective anchors for the metal ions [47]. These groups commonly consist of nitrogen-containing compounds such as amines, amides, and imines; oxygen-containing groups like hydroxyl, carboxylates, and ketones; or sulfur-containing groups like thiols and sulfides. An in-depth analysis of compound **5**–**12** indicates a requirement for these chelating groups. While the presence of a quinobenzothiazin core allows for possible interaction with metal ions, this core alone does not provide sufficient strength for chelation. In addition, the sulfonic acid group (-SO_3_H) is acidic and can form mild contact with metal ions, although it is not known for its ability to form strong chelating bonds. Thus, the compounds’ ability to operate as powerful chelating agents is greatly improved due to the need for these essential functional groups. The chlorine substituents present in compounds **11** and **7** and the sulfonic acid group exhibit electron-withdrawing effects. These effects modify the electron density surrounding possible metal-binding sites, reducing their favorability for interaction [48]. The decreased electron density reduces the attractive interactions between the chemicals and metal ions, hence reducing the chelation process. The lack of chelating activity in compounds can be elucidated by comparing their molecular structures and characteristics with those of the compounds examined by us earlier [29]. These studies exhibited significant chelating ability, attaining low IC_50_ values in their research. This indicates that functional groups and their spatial configurations in those compounds efficiently promote metal ion binding. In summary, according to our previous studies, it has been proven that diquinothiazine derivatives possess significant chelating properties. However, the unique chemical structure of the tested compounds, along with potentially insufficient experimental conditions, likely contributes to their lack of chelating activity. Ultimately, the compounds **5**–**12** exhibit characteristics that indicate they may be able to bind to metals. However, their inability to possess specific chelating groups and factors such as steric hindrance and electron-withdrawing effects can account for the observed lack of chelating activity. Nonetheless, distinct antioxidants may demonstrate differing levels of antioxidant efficacy owing to variations in their molecular architecture and modes of action. Although the compounds did not exhibit chelating ability, they may still have antioxidant capabilities capable of efficiently neutralizing reactive oxygen species (ROS) via other mechanisms.

#### 3.2.5. Pancreatic Lipase Esterase Assay

**Figure 3:**
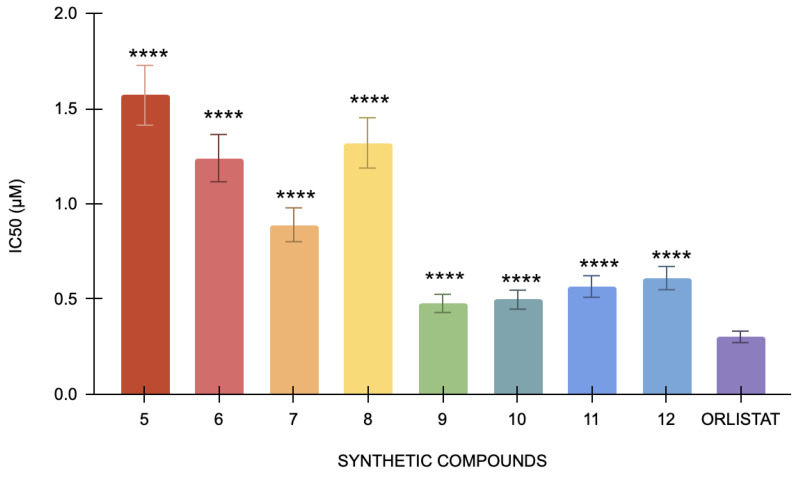
The pancreatic lipase inhibitory activity of compounds **5** to **12** against the standard, orlistat (*n* = 3). One-way ANOVA coupled with Dunnett’s multiple comparison test is represented by **** *p* < 0.0001 against orlistat.

The pancreatic lipase assay’s positive control, orlistat, has an IC_50_ value of 0.3 μM. By contrast, IC_50_ values for compounds **5**, **6**, **7**, **8**, **9**, **10**, **11**, and **12** were 1.57 μM, 1.24, 0.89 μM, 1.32 μM, 0.67 μM, 0.477 μM, 0.87 μM, and 0.566 μM, respectively (Figure 3). With an IC_50_ value of 0.477 μM, Compound **9** has the lowest value among the tested compounds. Conversely, compound **5** has the highest IC_50_ value of 1.57 μM, suggesting that it has the least inhibition in this study. Compared to orlistat, the compounds have demonstrated significant differences in their IC_50_ values, yielding a *p*-value < 0.0001. The differences in lipase inhibition among these synthetic compounds are statistically significant, suggesting that each compound exhibits a distinct level of efficacy compared to the positive control.

#### 3.2.6. Cholesterol Esterase Assay

**Figure 4:**
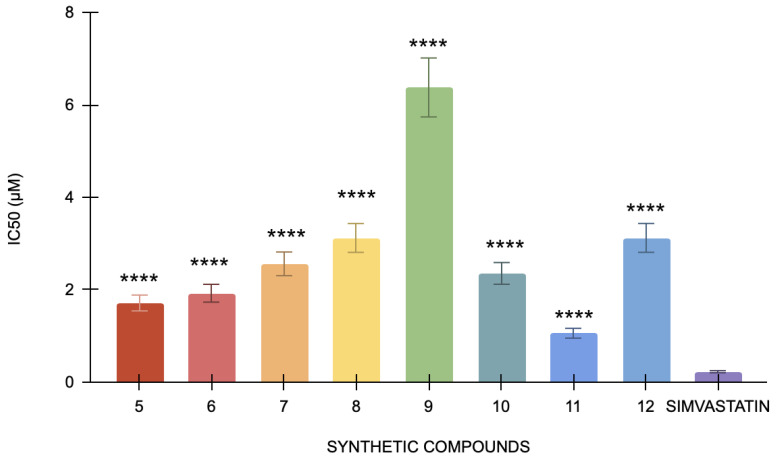
The cholesterol esterase inhibitory activity of compounds **5** to **12** against the standard, simvastatin (*n* = 3). One-way ANOVA coupled with Dunnett’s multiple comparison test is represented as **** *p* < 0.0001 against simvastatin.

In the cholesterol esterase assay, simvastatin, the positive control, has an IC_50_ of 0.22 μM. Specifically, compound **12** has the lowest IC_50_ value (1.05 μM), indicating that it is the most potent inhibitor of cholesterol esterase since it needs the least amount of concentration to 50% block the enzyme (Figure 4). Conversely, compound **9** has the highest IC_50_ value of 6.37 μM, indicating that it is less potent than the other compounds. The statistical analysis of the studied compounds against simvastatin showed a strong statistically significant difference (*p* < 0.0001). In this bioassay, compound **12** is the most powerful, and compound **9** is the least potent, indicating that each compound has considerably different cholesterol esterase inhibitory action when compared to simvastatin. It indicated that although simvastatin effectively inhibits cholesterol esterase, its potency is relatively lower than other compounds being tested. These results confirm that all the compounds are significantly better inhibitors of cholesterol esterase than simvastatin. Among these, compound **12** has shown the highest potency at the lowest concentration.

#### 3.2.7. Antiglycation of BSA-MGO

**Figure 5:**
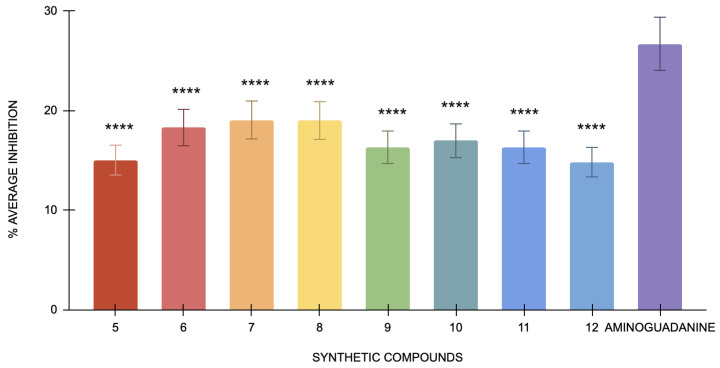
The antiglycation activity of compounds **5** to **12** and the standard, aminoguanidine, in the BSA-MGO model (*n* = 3). One-way ANOVA coupled with Dunnett’s multiple comparison test is represented by **** *p* < 0.0001 against aminoguanidine.

All eight compounds showed low percentages of inhibition (Figure 5). The low percentage of inhibition also affects the derivation of IC_50_ values. IC_50_ represents the inhibitor concentration necessary to attain 50% of its maximum effect. When the inhibition observed is minimal, it becomes challenging to determine a meaningful IC_50_ value because the inhibitor’s effect is not substantial enough to provide a reliable estimate. The lack of significant inhibition implies that the inhibitory effects of the compounds are either too weak or that the reaction conditions did not favor detecting such effects. This result suggests that the inhibitors tested had only a minimal impact on reducing MGO-induced glycation under the conditions used in this study. The low level of inhibition could be attributed to the intense reactivity of MGO, which may surpass the inhibitory effects of the compounds. Additionally, rapid reaction of MGO with BSA may limit the effectiveness of inhibitors in preventing glycation.

#### 3.2.8. Antiglycation of BSA-Glucose

**Figure 6:**
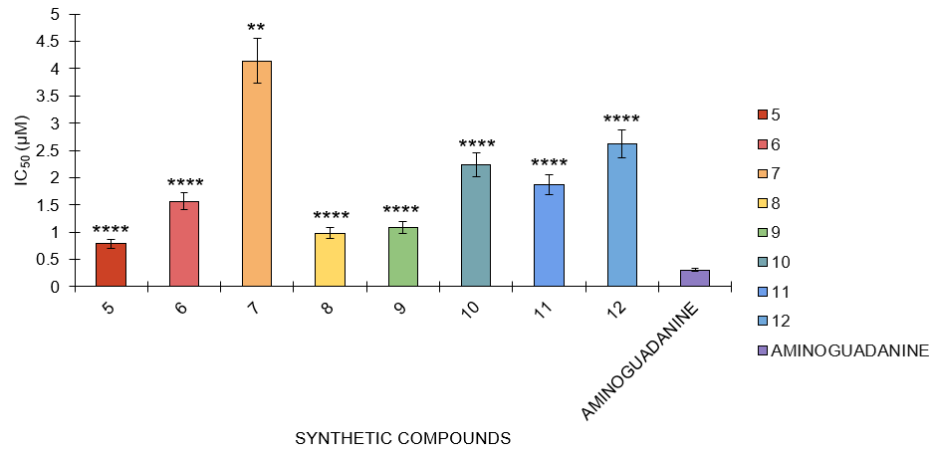
The antiglycation activity of compounds **5** to **12** against the standard, aminoguanidine, in the BSA-glucose model (*n* = 3). One-way ANOVA coupled with Dunnett’s multiple comparison test is represented by ** *p* < 0.01 and **** *p* < 0.0001 against aminoguanidine.

In the BSA-glucose assay, the following IC_50_ values were determined for several compounds: aminoguanidine had an IC_50_ value of 0.31 μM, while compounds **5**, **6**, **7**, **8**, **9, 10, 11,** and **12** had IC_50_ values of 0.79 μM, 1.57 μM, 4.13 μM, 0.98 μM, 1.09 μM, 2.24 μM, 1.89 μM, and 1.80 μM, respectively (Figure 6). Aminoguanidine has the smallest IC_50_ value, which means this compound is the most potent in this assay against the reaction because only the lowest concentration of this compound is needed to achieve an inhibition of 50%. Compounds **5** and **10** are also relatively potent but less potent than aminoguanidine. Of all the compounds, compound **7** has the highest value of IC_50_, which is 4.13 μM, indicating that among the tested compounds, it shows the poorest efficacy and hence requires a higher concentration to cause 50% inhibition.

The results showed that compound **10** had the lowest inhibition in the BSA-glucose test, whereas aminoguanidine had the highest. Changes in IC_50_ values show the relative efficiencies of compounds; the lower the IC_50_ value, the better the efficiency. The statistical analysis of all the tested compounds showed a strong statistically significant difference against aminoguanidine, with a *p*-value lower than 0.0001. This implies that each compound has a significantly different capacity for inhibition of the reaction compared to aminoguanidine. Despite their respective potencies, aminoguanidine is the strongest inhibitor, while the other compounds, particularly compound **10**, require higher concentrations to achieve comparable inhibition magnitudes. When put next to the other compounds tested, the statistical significance of aminoguanidine brings out the comparative efficacy.

### 3.3. Docking Studies

Table 2 shows the binding energies of the synthetic compounds to the pancreatic lipase and cholesterol esterase, which indicates the interaction between energies and enzymes. For pancreatic lipase, it ranged from –8.5 kcal/mol (**6**) to –8.9 kcal/mol (**9**). Notably, compounds **5** and **9** share the same binding energy against pancreatic lipase (−8.9 kcal/mol). The compound **6** shows the lowest binding energies for pancreatic lipase, indicating relatively weaker interactions. In contrast, all the compound binding energies were slightly lower when docked into cholesterol esterase. Compound **9** again showed the strongest binding energy for cholesterol esterase. Compounds **7** and **12** show the weakest binding energies with cholesterol esterase (−7.4 kcal/mol). Overall, compound **9** shows a promising compound in terms of binding energies with both enzymes.

Table 2 summarizes the binding affinities of the compounds with pancreatic lipase and cholesterol esterase, the key enzymes responsible for lipid digestion and metabolism, as obtained from molecular docking. Overall, the variations in the binding energies of the compounds may be influenced by their substitutions at position x and chain length (n). Among all the tested compounds, compound **9** exhibits the lowest binding energy for both pancreatic lipase (−8.9 kcal/mol) and cholesterol esterase (−8.0 kcal/mol), indicating the strongest binding to both enzymes. This suggests that the structural features of this compound may enhance its stability within the active sites of both enzymes.

**Figure 7:**
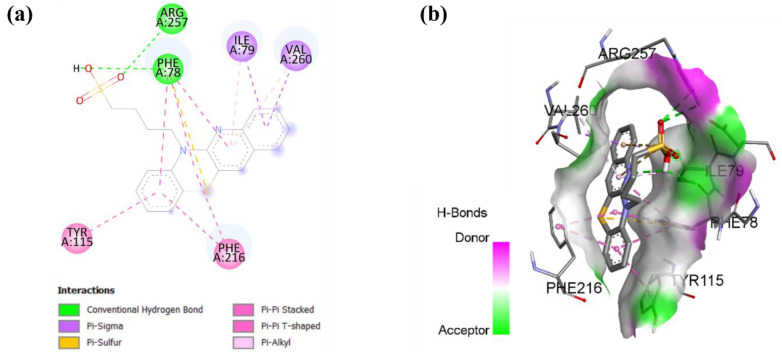
Molecular docking showing (**a**) 2D protein–ligand and (**b**) 3D protein–ligand interactions of compound **9** with pancreatic lipase.

As shown in Figure 7, molecular docking analysis revealed the interactions underlying compound **9** binding within pancreatic lipase. Compound **9** forms hydrogen bonds with residues ARG 257 and PHE 78 and is further stabilized by multiple hydrophobic interactions with residues ILE 79, VAL 260, PHE 216, and TYR 115. These multiple stabilizing interactions suggest that this compound can effectively block substrate access to the pancreatic lipase catalytic site, potentially reducing cholesterol metabolism [49].

**Figure 8:**
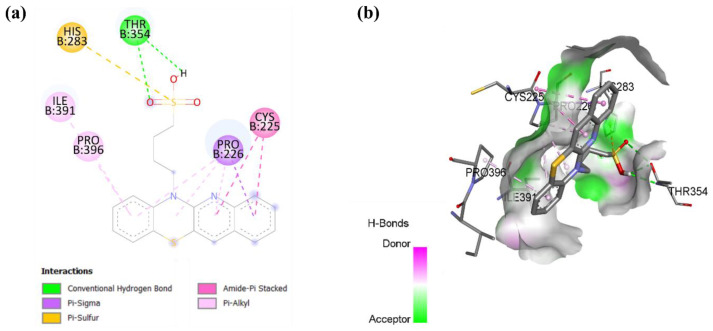
Molecular docking showing (**a**) 2D protein–ligand and (**b**) 3D protein–ligand interactions of compound **9** with cholesterol esterase.

In cholesterol esterase, compound **9** forms a hydrogen bond with residue THR 354 and a pi-sulfur bond with residue HIS 283, as shown in Figure 8. The interactions are further stabilized by multiple hydrophobic interactions, including a pi-sigma bond with residue PRO 226, an amide-pi stacked bond with residue CYS 225, and a pi-alkyl bond with residues PRO 396 and ILE 391. These findings suggest that compound **9** may have the potential as an inhibitor of cholesterol esterase, which could be beneficial in reducing cholesterol metabolism and addressing hypercholesterolemia-related conditions [50].

The comparison of compounds with different X substituents highlights the impact of the functional group at this position. For instance, halogen substitutions in compound **6** and compound **7** with fluorine and chlorine resulted in slightly weaker binding affinities compared to compound **9**. In addition, compounds **8** and **12**, with X = SCH_3_, consistently showed higher binding affinities compared to compound **9**, which may indicate that the bulkiness of this group hinders optimal interactions within the active site of both enzymes.

Additionally, chain length also influences the binding affinity. Compounds with *n* = 4, such as compound **9**, generally exhibited stronger binding than compound **5** with *n* = 3 for both enzymes. This pattern suggests that an extended chain length may allow for better interactions within the active site and potentially enhance hydrophobic interactions for favorable binding.

## 4. Discussion

The aim of our studies was to synthesize eight new phenothiazine analogs obtained by modifying the phenothiazine system with a quinoline ring and introducing substituents containing a sulfonic acid fragment to the thiazine nitrogen atom. We obtained the new quinobenzothiazines in a two-step synthesis based on 2,2′-dichloro-3,3′-diquinolyl disulfide as a substrate for the synthesis of N-*H* quinobenzothiazines **1**–**4**. These previously developed and described quinobenzothiazines were reacted with propane- or butanesultone to obtain the title compounds **5**–**12**. The structure of compounds **5**–**12** was confirmed by ^1^H NMR, ^13^C NMR, and HRMS analyses. The newly obtained quinobenzothiazines were evaluated for their toxicity, antiglycation capacity, antioxidant properties, and potential to combat hyperlipidemia.

The MTT assay is used to gauge the toxicity of drug molecules on the viability of the cells. This experiment shows that tested compounds **5**–**12** were not toxic to the cells at the tested concentrations (Figure 1). Several researchers have used this assay to evaluate the toxicity of many drug molecules and phytochemicals. The experiment suggests that compounds **5**–**12** are safe to be used as drug molecules for the treatment of diseases. However, further in vivo evaluation is needed to further buttress this claim.

Naturally occurring during cellular respiration and metabolism, reactive oxygen species (ROS) are extremely reactive chemicals [51]. Once thought to be harmful solely because they may damage cells, studies have shown their complex role in the development of obesity and related metabolic disorders [52]. ROS plays a vital function in regulating a number of physiological processes that lead to the development of adipose tissue and the disturbance of metabolic equilibrium. It significantly alters mitochondrial function, which is linked to obesity. Adipocyte function and lipid metabolism can be greatly impacted by the interplay between reactive oxygen species and mitochondrial activity, which can ultimately lead to weight gain. Additionally, it plays a vital role in adipogenesis, converting preadipocytes into mature fat cells [53]. ROS promotes fat accumulation by controlling genes essential for adipocyte growth and lipid storage. Furthermore, there is proof that reactive oxygen species (ROS) influence hypothalamic neurons, which in turn may play a part in appetite regulation. Through controlling neurons that govern hunger, ROS may have an impact on eating and energy balance, which in turn influences weight and fat storage [54]. Chronic hypernutrition, high-fat or high-carbohydrate meals, SFA, and trans-fatty acid consumption are examples of overnutrition that increases oxidative stress across a variety of intricate biochemical pathways [55]. These include the polyol/hexosamine pathway, oxidative phosphorylation, glyceraldehyde autooxidation, PKC activation, and Nox-dependent NADPH oxidase [56]. By altering the way white adipose tissue is laid down and the regulation of food intake, this chronic oxidative stress subsequently triggers the activation of important signaling pathways that can initiate and sustain the development of obesity [57]. Studies on oxidative stress in animal models and cell cultures have demonstrated that oxidative stress can promote the growth of pre-adipocytes, aid in adipocyte differentiation, and increase the size of mature adipocytes [58]. By influencing certain hypothalamic neurons that control appetite and satiety behaviors, ROS, an essential mediator of oxidative stress, appears to affect weight homeostasis [59]. The intricate relationships between obesity and oxidative stress highlight the different ways that redox abnormalities fuel the emergence of obesity. By interfering with adipose tissue physiology (increased leptin production) and hormone control (ghrelin secretion), oxidative stress exacerbates obesity and metabolic comorbidities [60]. The process of turning pre-adipocytes into adipocytes, known as adipogenesis, is delicate and intricate, and reactive oxygen species (ROS) have various effects on it [61]. By upsetting the equilibrium of redox processes, an excessive amount of ROS prevents this conversion [62]. However, at low concentrations, ROS do contribute to the signaling pathways that support cell development. Efficient regulation of ROS is necessary to control the primary adipogenic transcription factors and pathways. Masenga et al. (2023) claim that ROS molecules have an impact on the transcription of genes related to differentiation, lipid metabolism, and insulin sensitivity [63]. The primary regulator of adipogenesis, peroxisome proliferator-activated receptor gamma (PPARγ), may be activated by mild concentrations of ROS. According to Wu, Li and Shen, PPARγ promotes cell differentiation by regulating the expression of genes involved in fat cell formation, uptake, and accumulation [64]. To start adipogenesis, ROS must activate PPARγ [65]. However, oxidative stress, which can damage the process by upsetting intricate redox signaling networks, can be brought on by an excess of reactive oxygen species (ROS) [66]. It has been shown that elevated reactive oxygen species (ROS) inhibit PPARγ and genes involved in adipogenesis, the process by which fat cells form, leading to defective adipocyte development and disturbed metabolism [67]. The body’s overall energy balance is impacted by reactive oxygen species (ROS), which have an effect on cellular energy generation and metabolism [68]. Adipose tissue growth, which is marked by an increase in the quantity and size of fat cells, is associated with this persistent, mild inflammation. ROS may exacerbate metabolic problems linked to obesity, including insulin resistance and high cholesterol [63]. Additionally, it plays a vital role in adipogenesis, converting preadipocytes into mature fat cells [53]. ROS promotes fat accumulation by controlling genes essential for adipocyte growth and lipid storage. Furthermore, there is proof that reactive oxygen species (ROS) influence hypothalamic neurons, which in turn may play a part in appetite regulation. Through controlling neurons that govern hunger, ROS may have an impact on eating and energy balance, which in turn influences weight and fat storage [54]. According to a number of research studies that have been cited, ROS has been positively associated with hyperlipidemia. Tested compounds may have an antihyperlipidemia and anti-glycation effect because of their capacity to block ROS. Numerous pathways, including the polyol pathway, activation of aldose reductase (ALR2), and the first rate-limiting enzyme of the polyol pathway, have been implicated in the increased generation of AGEs as a result of oxidative stress generation and hyperlipidemia [69]. Oxidative stress speeds up the production of advanced glycation end products (AGE), according to several studies. Particularly in cases of hyperglycemia, AGEs have the ability to produce reactive oxygen species and hasten the glycation of proteins [70]. According to earlier studies, a weak antioxidant defense system and an insulin-resistant condition are caused by increased free radical formation through enhanced glycolysis, activated sorbitol, autoxidation of glucose, and non-enzymatic protein glycation [71]. AGEs are compounds made up of reduced sugars, lipids, proteins’ amino groups, and nucleic acids. Numerous free radicals, carbonyl species, and reactive dicarbonyl species are produced during the glycation process’s beginning and propagation phases. The most reactive is methylglyoxal (MG), which might influence normal physiological activities by causing dicarbonyl stress [69]. One important precursor of AGE is 3-deoxyglucosone, which is a byproduct of the polyol pathway. The external source of AGEs that is proportionate to elevated blood levels of AGE is diet, which is high in fat and protein [72]. The rate of AGE creation, which is influenced by oxidative stress and reducing sugars, and the rate of clearance, which is influenced by the glyoxalase system’s activity, dictate how many AGEs are present. Reactive carbonyl compounds can be detoxified by glyoxalase I (Glo I) [73].

AGE-inhibiting compounds also function as organic scavengers of oxidative stress. Aldose reductase inhibition, reactive carbonyl species scavenging, and antioxidant capacity are some of the mechanisms that underpin the tactics used to limit the production of AGE [41]. In order to prevent oxidative stress and hasten the formation of AGE, antioxidant activities are crucial. The majority of substances with antioxidant capacity block AGEs through a variety of mechanisms, according to numerous studies. According to Jariyapamornkoon et al., some proposed processes include metal ion chelation, carbonyl trapping, reducing power, suppression of lipid peroxidation, and radical scavenging [74]. Very effective in vitro suppression of BSA-glucose and MGO-BSA glycation was demonstrated by alkylsulfonic acids with quinobenzothiazinyl substituents. Blocking free radicals may have this impact by lowering oxidative stress and reducing the synthesis of reactive carbonyl and dicarbonyl groups. This action might have helped test alkylsulfonic acids with quinobenzothiazinyl substituents that inhibit pancreatic lipase and cholesterol esterase, which in turn led to the antihyperlipidemia seen in this study. Numerous studies have demonstrated that several current therapies for hyperlipidemia also have antioxidative qualities that lower the development of AGE. By inhibiting the development of AGEs, atorvastatin demonstrated a reduction in serum AGEs as well as a reduction in RAGE expression in carotid artery plaques [75]. Statins and a number of antihypertensive and antidiabetic medications also indirectly reduce AGEs. There have been reports of the therapeutic benefits of a number of novel AGE/ALE inhibitors in preventing nephropathy and dyslipidemia in rats with diabetes caused by streptozotocin (STZ). By blocking metal-catalyzed processes, reactive oxygen species (OH radical), reactive carbonyl species (methylglyoxal and glyoxal), and AGE-protein cross-linking reactions, LR-90 has been shown to possess general antioxidant qualities. By preventing AGE buildup, RAGE protein expression, and protein oxidation in the diabetic kidney, the results show that LR-90 is a bioefficacious treatment for nephropathy and hyperlipidemia in diabetic mice [76].

## 5. Conclusions

The increased production of AGEs due to oxidative stress generation and hyperlipidemia has been linked to a number of pathways, including the polyol pathway, activation of aldose reductase (ALR2), and the polyol pathway’s first rate-limiting enzyme. Alkylsulfonic acids with quinobenzothiazinyl substituents (**5**–**12**) have shown to possess strong antiglycation through the inhibition of BSA-glucose and BSA-MGO glycation. Compounds **11** and **12** showed the highest levels of activity. Both of these new alkylsulfonic acids with quinobenzothiazinyl substituents contain a four-carbon linker between the acid group and the thiazine carbon atom. Compound **11** additionally contains a chloro substituent, and compound **12** a methylthiol substituent at the 9-position of the quinobenzothiazine system. The least active, although in studies more active than the reference drugs, were derivatives without a substituent in the benzene ring of the quinobenzothiazine system. Antihyperlipidemia via the inhibition of cholesterol esterase and pancreatic lipase. All these effects may be through its antioxidant property, which has been established through several reports.

## Figures and Tables

**Table 1:** Percentage ROS generation of each compound in differentiated 3T3-L1 adipocytes.

Compound	% ROS Generation at Each Concentration				
	0.02 μM	0.06 μM	0.1 μM	0.2 μM	0.25 μM
**5**	208.69 ± 13.07 ^a^	142.87 ± 15.43 ^b^	76.83 ± 0.0 ^c^	39.72 ± 6.30 ^c^	39.24 ± 0.0 ^c^
**6**	137.15 ± 9.37 ^a^	126.66 ± 2.43 ^a^	88.31 ± 3.13 ^b^	69.68 ± 7.12 ^c^	63.73 ± 1.04 ^c^
**7**	143.18 ± 10.65 ^a^	113.48 ± 10.27 ^a^	33.27 ± 6.16 ^b^	15.63 ± 2.74 ^b^	14.96 ± 1.25 ^b^
**8**	149.62 ± 6.71 ^a^	101.04 ± 7.69 ^b^	71.47 ± 2.98 ^c^	62.94 ± 4.59 ^c^	50.89 ± 2.85 ^d^
**9**	169.62 ± 13.81 ^a^	151.45 ± 25.13 ^ab^	151.54 ± 32.18 ^ab^	61.36 ± 1.58 ^c^	26.38 ± 1.73 ^c^
**10**	103.85 ± 1.17 ^a^	74.74 ± 2.90 ^b^	69.81 ± 5.23 ^b^	52.56 ± 4.14 ^c^	47.16 ± 3.71 ^c^
**11**	104.90 ± 5.31 ^a^	35.75 ± 11.03 ^c^	46.64 ± 6.13 ^bc^	75.96 ± 2.21 ^ab^	57.18 ± 1.66 ^bc^
**12**	119.82 ± 5.12 ^a^	102.85 ± 1.47 ^b^	97.18 ± 2.61 ^b^	80.38 ± 1.72 ^c^	60.12 ± 1.74 ^d^

Two-way ANOVA coupled with Tukey’s post hoc test is represented by the superscript letters, where groups with the same letters are non-significant.

**Table 2:** Binding energies of compounds **5** to **12** against pancreatic lipase and cholesterol esterase.

Compounds	Binding Energies (kcal/mol)	
	Pancreatic Lipase	Cholesterol Esterase
**5**	−8.9	−7.9
**6**	−8.5	−7.5
**7**	−8.6	−7.4
**8**	−8.6	−7.8
**9**	−8.9 *	−8.0 *
**10**	−8.7	−7.6
**11**	−8.8	−7.9
**12**	−8.6	−7.4

The compound with the strongest binding energy for both pancreatic lipase and cholesterol esterase is marked with an asterisk (*).

## Data Availability

Data are contained within the article and Supplementary Materials.

## Supplementary Materials

The following supporting information can be downloaded at: https://www.mdpi.com/article/10.3390/antiox14040464/s1, ^1^H NMR and ^13^C NMR spectra and HR MS of compounds **5**–**12**.
