# Supplementary material for: A Study of Antioxidant, Antihyperlipidemic, and Anti-Glycation Effects of Alkylsulfonic Acids with Quinobenzothiazinyl Substituents: In Vitro and In Silico Investigations
Source: Antioxidants (Basel). 2025 Apr 12;14(4):464. doi: 10.3390/antiox14040464 (PMC12024154; doi:10.3390/antiox14040464)
Supplement: Supplementary file 1 [file antioxidants-14-00464-s001.zip › antioxidants-3548466-supplementary.pdf]

A Study of Antioxidant, Antihyperlipidemia and Anti-glycation Effect of Alkilosulfonic Acids with Quinobenzothiazinly Substituents: *In Vitro* and *In Silico* Investigation

Kirthani Anamalay [1], Lee Qiao Er [1], Abbirami Balachandran [1], Patrick Nwabueze Okechukwu [1,2*], Beata Morak-Młodawska [3], Merell P. Billacura [4], Charlie A. Lavilla Jr. [5], Anis Najwa Abdul Rani [6], Anand Gaurav [6], Adam Konefał [7], and Małgorzata Jeleń [3*]

1. Department of Biotechnology, Faculty of Applied sciences, Ucsi University, No.1 Jalan Menara Gading, UCSI Heights (Taman Connaught), Cheras, 56000 Kuala Lumpur, Malaysia, kirthani1296@gmail.com (K.A.), qiaoer1007@gmail.com (L.Q.R.), abbirami18@gmail.com (A.B.), patrickn@ucsiuniversity.edu.my (P.O.N.)
2. Department of Pharmacology, Faculty of Pharmacy, Capital City University Kano- Nigeria, patricknwa@ccuk.edu.ng (P.N.O.)
3. Department of Organic Chemistry, Faculty Of Pharmaceutical Sciences, Medical University of Silesia, Jagiellońska, Str. 4, 41-200 Sosnowiec, Poland, bmlodawska@sum.edu.pl (B.M.M.), manowak@sum.edu.pl (M.J.).
4. Chemistry Department, College of Science & Mathematics, Mindanao State University-Iligan Institute of Technology, Iligan City 9200, Lanao del Norte, Philippines, merell.billacura@msumain.edu.ph (M.P.B.)
5. Department of Chemistry, College of Natural Sciences and Mathematics, Mindanao State University-Main Campus, Marawi City 9700, Lanao del Sur, Philippines, charliejr.lavilla@g.msuiit.edu.ph (C.A.L.).
6. Faculty of Pharmaceutical Sciences, UCSI University, 56000 Cheras, Kuala Lumpur, Malaysia, anisnajwabintiabdulrani@gmail.com (A.N.A.R.), anand.pharma@gmail.com (A.G.).
7. Institute of Physics, University of Silesia in Katowice, 40-007 Katowice, Poland, adam.konefal@us.edu.pl, (A.K.).

* Correspondence: patrickn@ucsiuniversity.edu.my (P.N.O.); manowak@sum.edu.pl (M.J.) Tel.: +603-9101-8880 (P.N.O.); +48-32-364-16-04 (M.J.)

Content:

^{1}H NMR and ^{13}C NMR spectra and HR MS of compounds **5 - 12**.

Compound 5

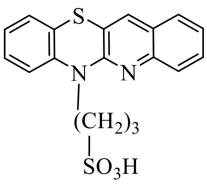

S
N
N
(CH2)3
SO3H

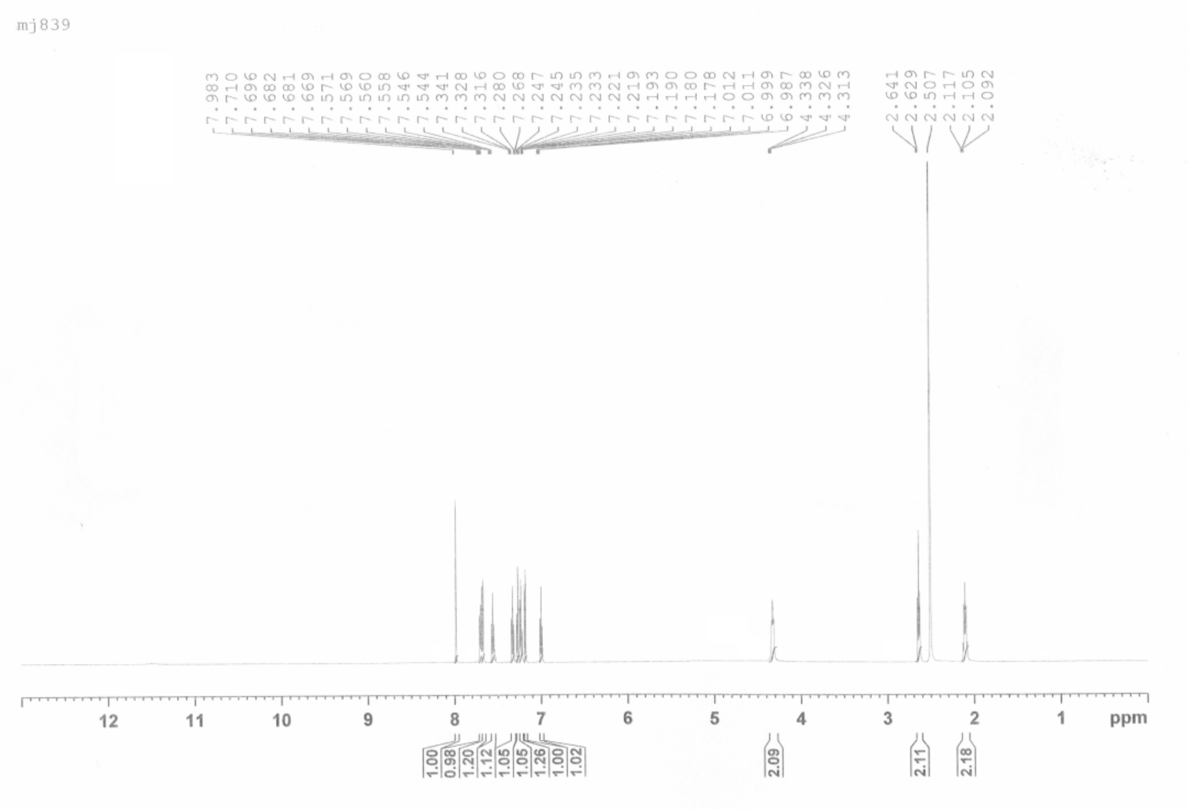

mj839
7.983
7.710
7.696
7.682
7.681
7.669
7.571
7.569
7.560
7.558
7.546
7.544
7.341
7.328
7.316
7.280
7.268
7.247
7.245
7.235
7.233
7.221
7.219
7.193
7.190
7.180
7.178
7.012
7.011
6.999
6.987
4.338
4.326
4.313
2.641
2.629
2.507
2.117
2.105
2.092
12
11
10
9
8
7
6
5
4
3
2
1
ppm
1.00
0.98
1.20
1.12
1.05
1.05
1.26
1.00
1.02
2.09
2.11
2.18

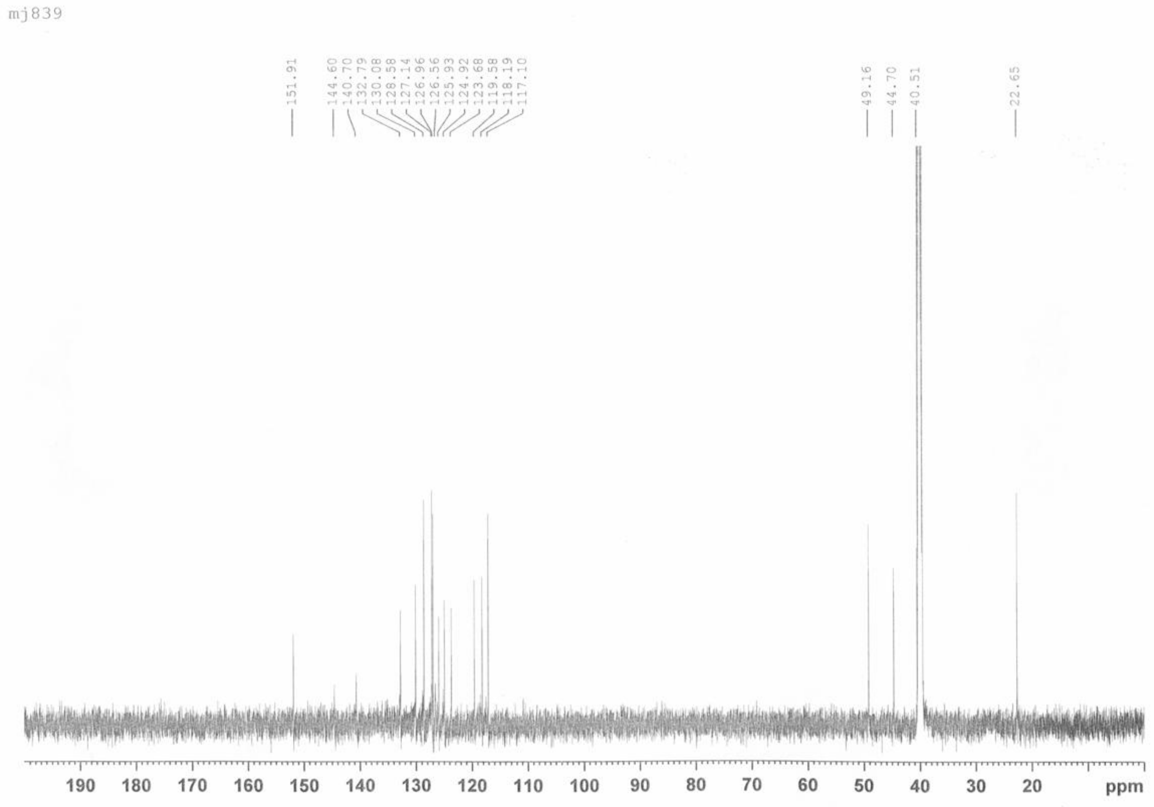

Acquisition Parameter

Source Type	ESI	Ion Polarity	Positive	Set Nebulizer	0.3 Bar
Focus	Active	Set Capillary	4000 V	Set Dry Heater	200 °C
Scan Begin	100 m/z	Set End Plate Offset	-500 V	Set Dry Gas	3.0 l/min
Scan End	1000 m/z	Set Charging Voltage	2000 V	Set Divert Valve	Source
		Set Corona	0 nA	Set APCI Heater	0 °C

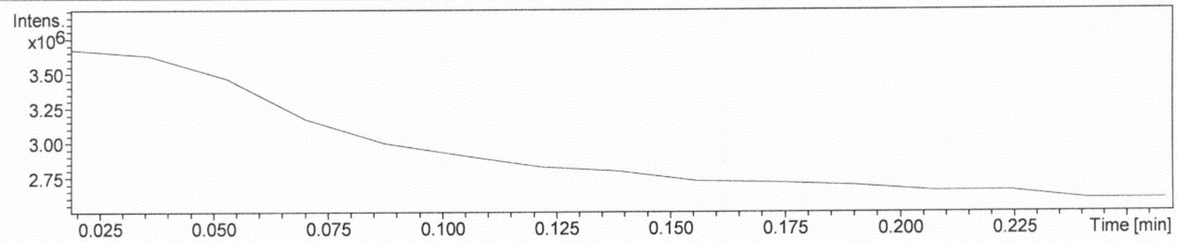

+MS, 0.1-0.2min #7-11

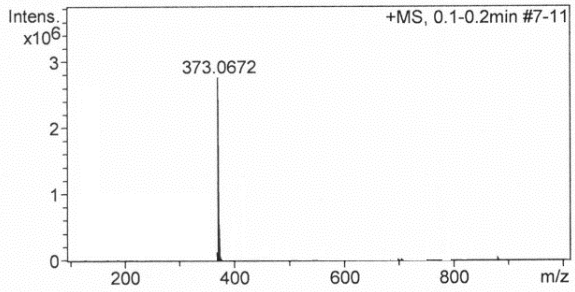

#	m/z	Res.	S/N	I	I %	FWHM
1	373.0672	37947	39399.3	2755405	100.0	0.0098

Compound 6

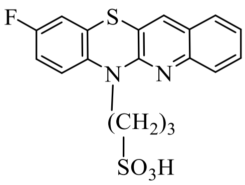
F
S
N
N
(CH2)3
SO3H

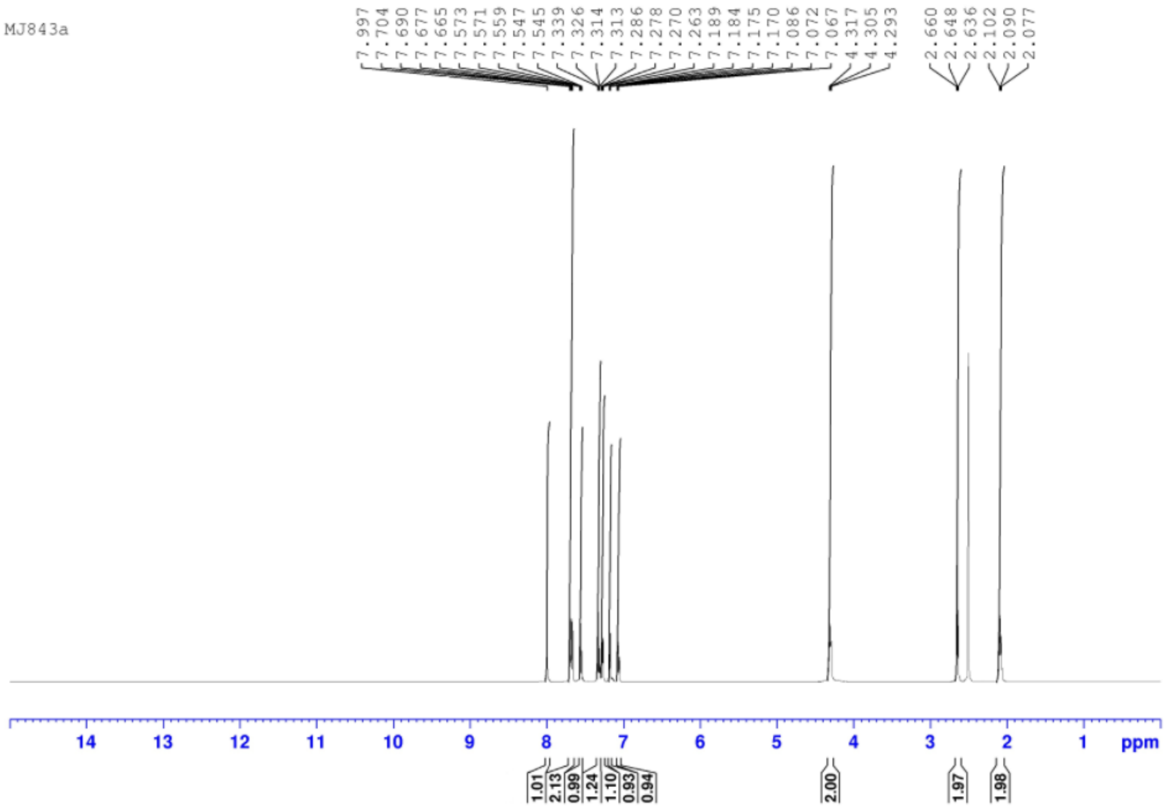
MJ843a
7.997
7.704
7.690
7.677
7.665
7.573
7.571
7.559
7.547
7.545
7.339
7.326
7.314
7.313
7.286
7.278
7.270
7.263
7.189
7.184
7.175
7.170
7.086
7.072
7.067
4.317
4.305
4.293
2.660
2.648
2.636
2.102
2.090
2.077
14
13
12
11
10
9
8
7
6
5
4
3
2
1
ppm
1.01
2.13
0.99
1.24
1.10
0.93
0.94
2.00
1.97
1.98

MJ843a

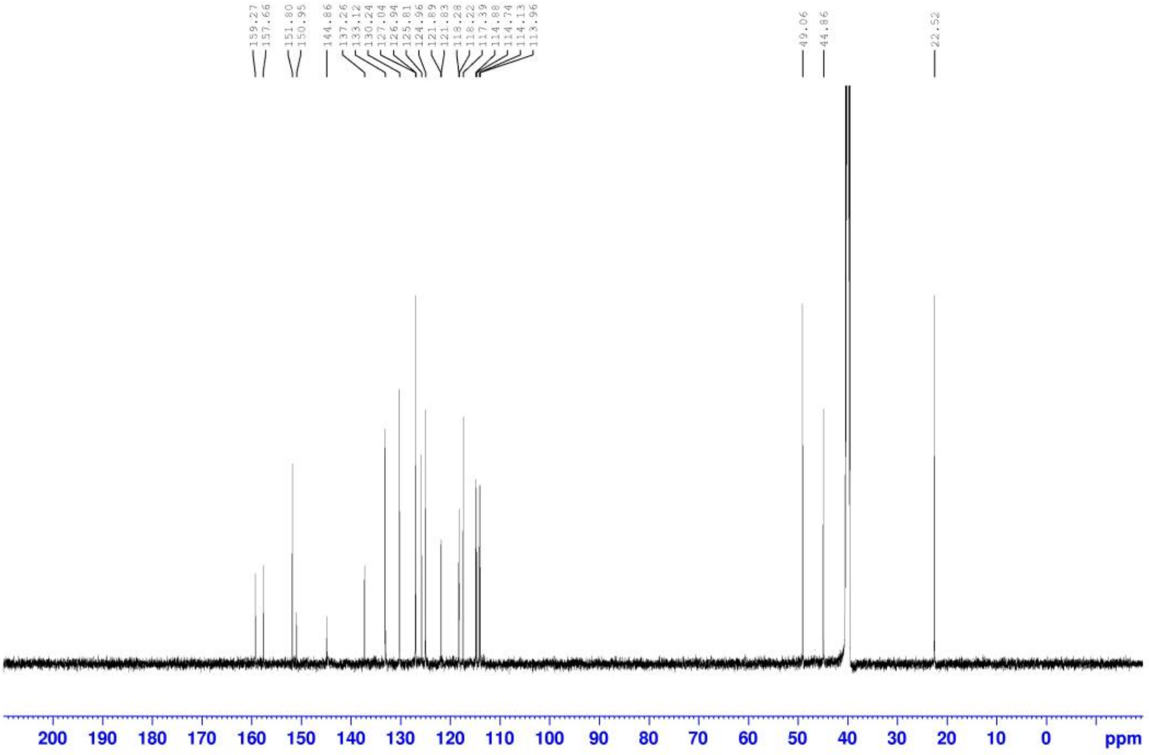

Acquisition Parameter

Source Type	ESI	Ion Polarity	Positive	Set Nebulizer	0.3 Bar
Focus	Active	Set Capillary	4000 V	Set Dry Heater	200 °C
Scan Begin	100 m/z	Set End Plate Offset	-500 V	Set Dry Gas	3.0 l/min
Scan End	1000 m/z	Set Charging Voltage	2000 V	Set Divert Valve	Source
		Set Corona	0 nA	Set APCI Heater	0 °C

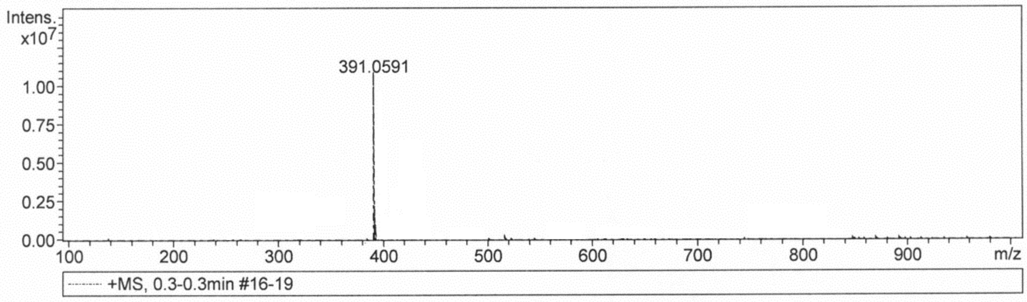

#	m/z	Res.	S/N	I	I %	FWHM
1	391.0591	42709	30653.7	10588725	100.0	0.0092

Compound 7

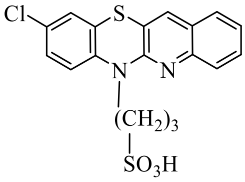

Cl
S
N
N
(CH2)3
SO3H

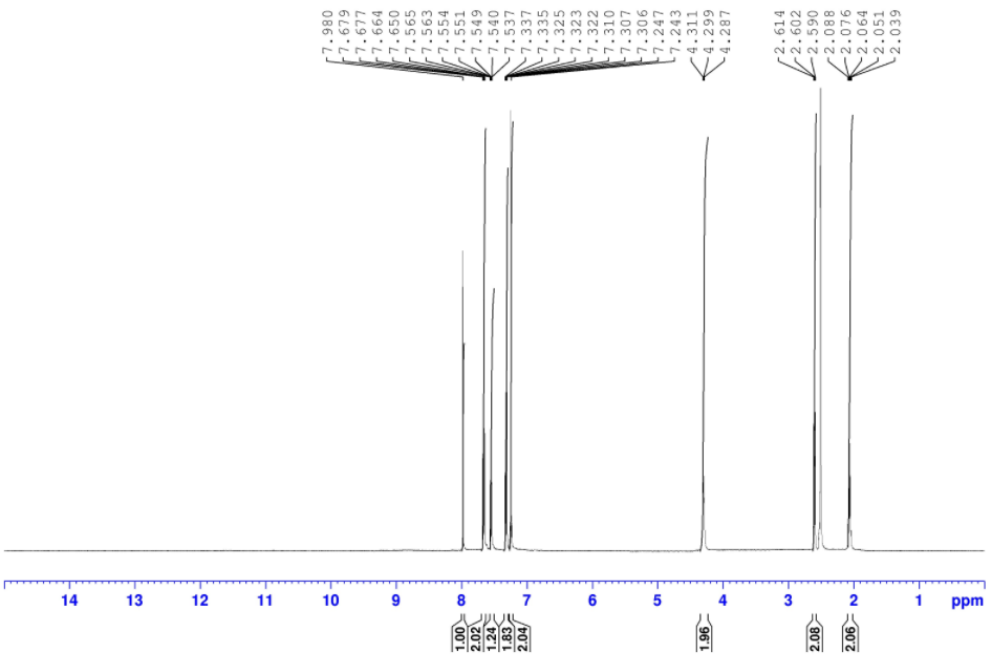

MJ840
7.980
7.679
7.677
7.664
7.650
7.565
7.563
7.554
7.551
7.549
7.540
7.537
7.337
7.335
7.325
7.323
7.322
7.310
7.307
7.306
7.247
7.243
4.311
4.299
4.287
2.614
2.602
2.590
2.088
2.076
2.064
2.051
2.039
14
13
12
11
10
9
8
7
6
5
4
3
2
1
ppm
1.00
2.02
1.24
1.83
2.04
1.96
2.08
2.06

MJ840

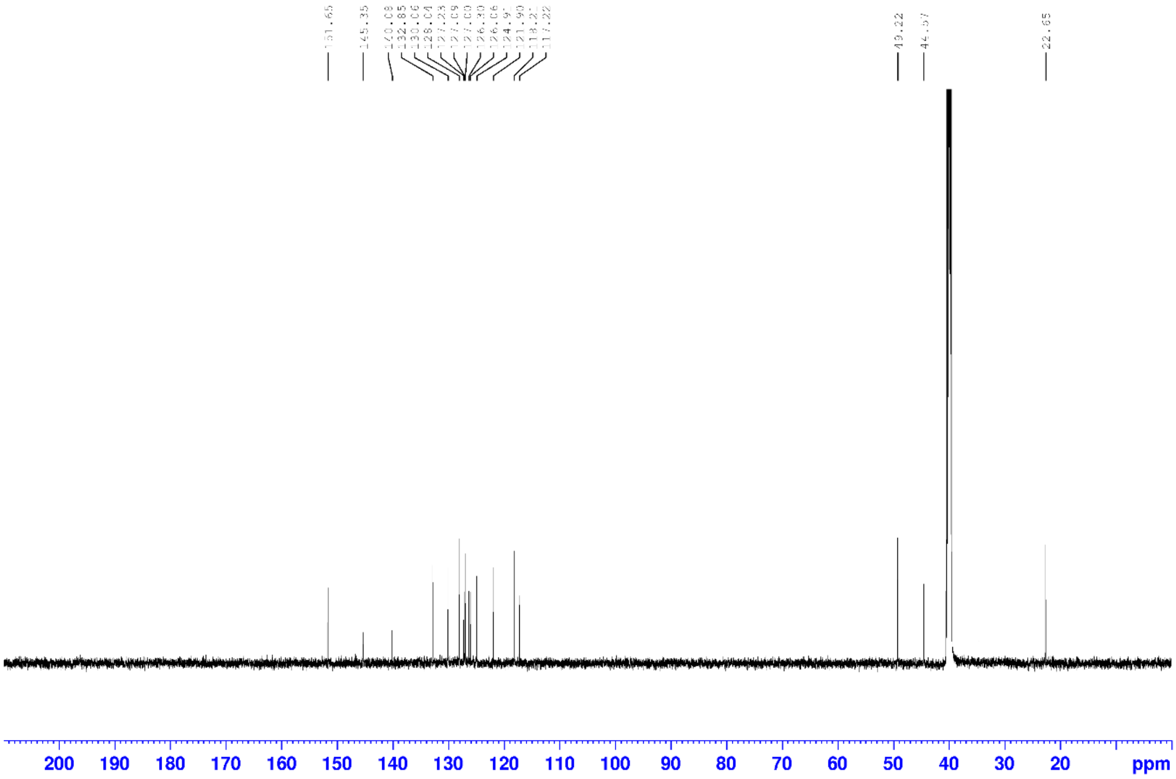

Acquisition Parameter

Source Type	ESI	Ion Polarity	Positive	Set Nebulizer	0.3 Bar
Focus	Active	Set Capillary	4000 V	Set Dry Heater	200 °C
Scan Begin	100 m/z	Set End Plate Offset	-500 V	Set Dry Gas	3.0 l/min
Scan End	1000 m/z	Set Charging Voltage	2000 V	Set Divert Valve	Source
		Set Corona	0 nA	Set APCI Heater	0 °C

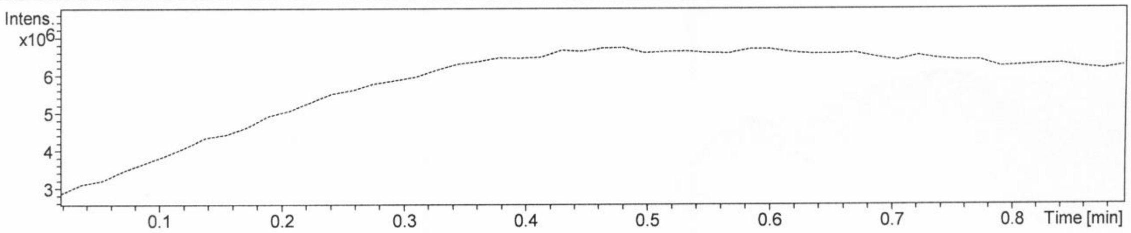

+MS, 0.3-0.7min #15-41

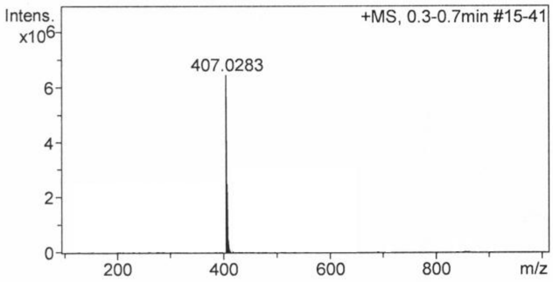

#	m/z	Res.	S/N	I	I %	FWHM
1	407.0283	42156	39128.8	6429701	100.0	0.0097
2	409.0255	36712	15041.4	2498580	38.9	0.0111

Compound 8

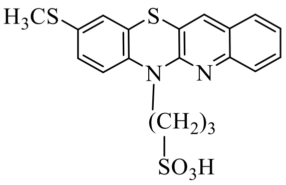
H3CS
S
N
N
(CH2)3
SO3H

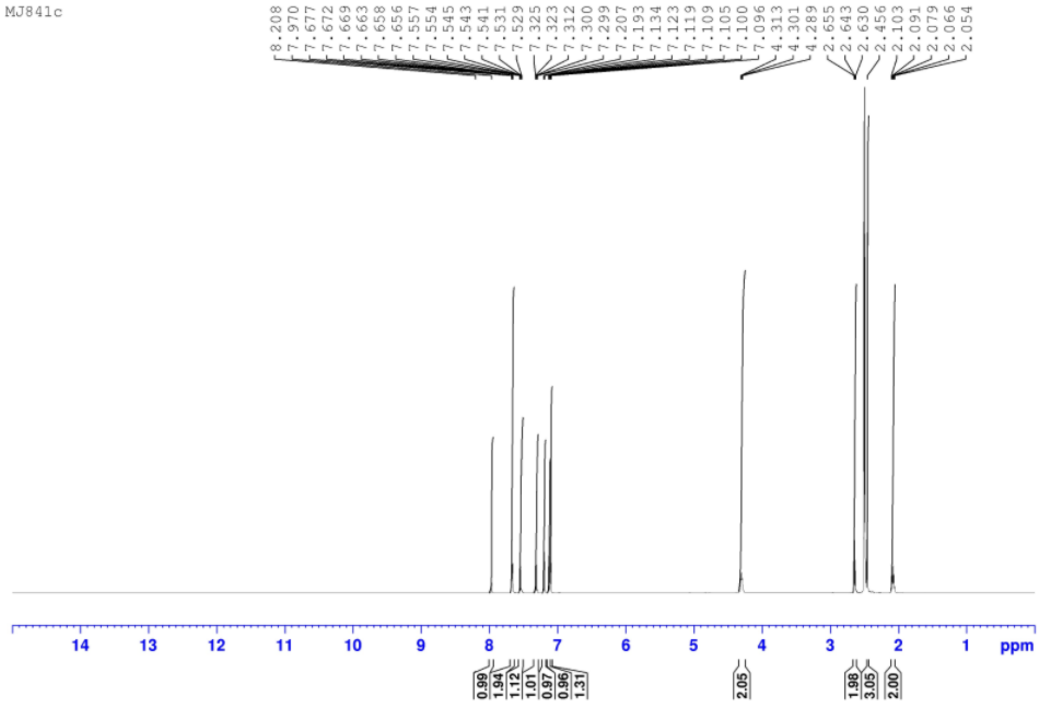
MJ841c
8.208
7.970
7.677
7.672
7.669
7.663
7.658
7.656
7.557
7.554
7.545
7.543
7.541
7.531
7.529
7.325
7.323
7.312
7.300
7.299
7.207
7.193
7.134
7.123
7.119
7.109
7.105
7.100
7.096
4.313
4.301
4.289
2.655
2.643
2.630
2.456
2.103
2.091
2.079
2.066
2.054
14
13
12
11
10
9
8
7
6
5
4
3
2
1
ppm
0.99
1.94
1.12
1.01
0.97
0.96
1.31
2.05
1.98
3.05
2.00

MJ841c

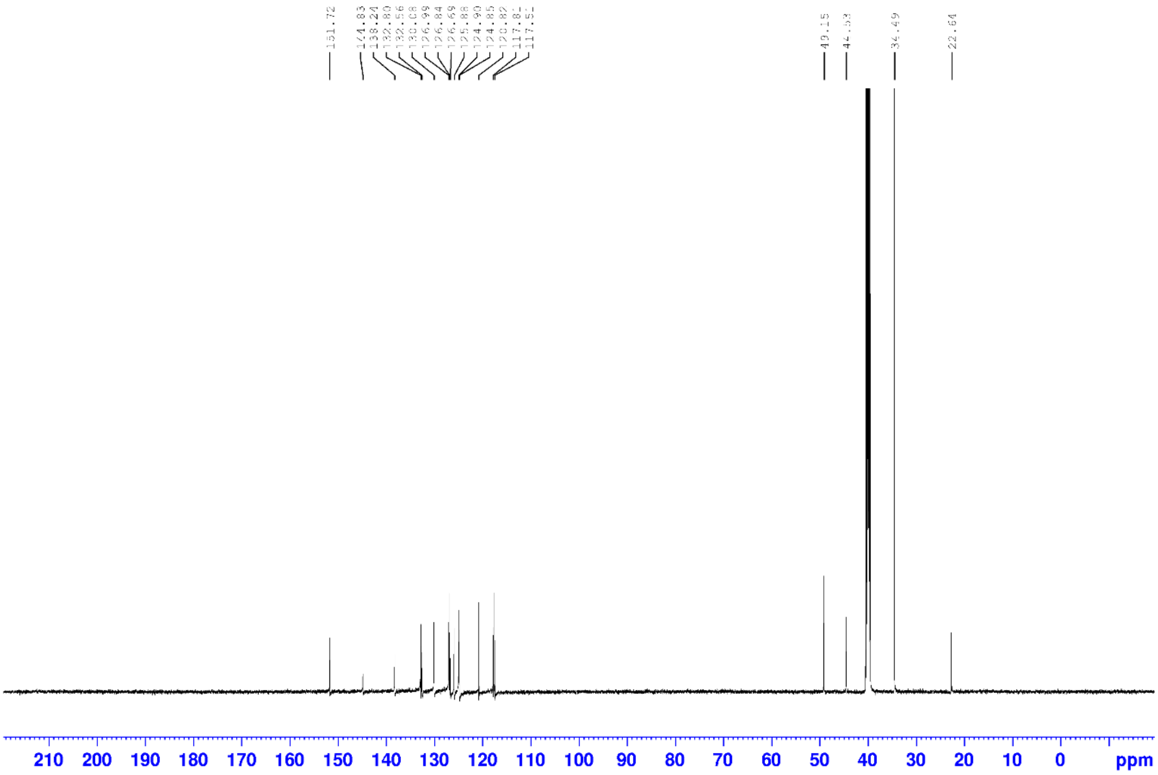

Acquisition Parameter

Source Type	ESI	Ion Polarity	Positive	Set Nebulizer	0.3 Bar
Focus	Active	Set Capillary	4000 V	Set Dry Heater	200 °C
Scan Begin	100 m/z	Set End Plate Offset	-500 V	Set Dry Gas	3.0 l/min
Scan End	1000 m/z	Set Charging Voltage	2000 V	Set Divert Valve	Source
		Set Corona	0 nA	Set APCI Heater	0 °C

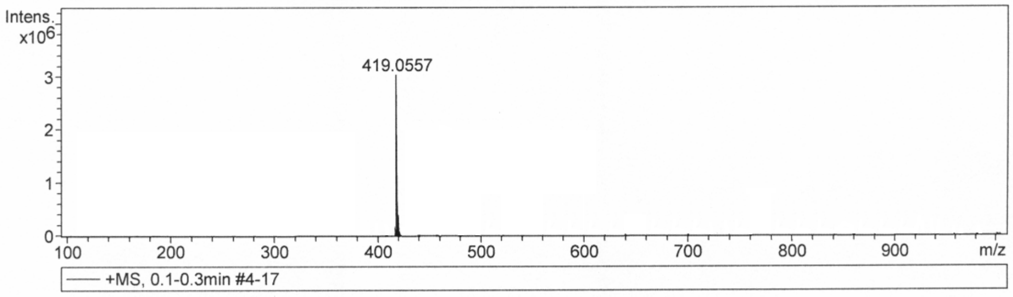

#	m/z	Res.	S/N	I	I %	FWHM
1	419.0557	37841	14547.0	3022295	100.0	0.0111
2	420.0586	28080	3137.3	657919	21.8	0.0150

Compound 9

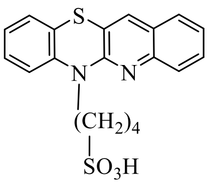

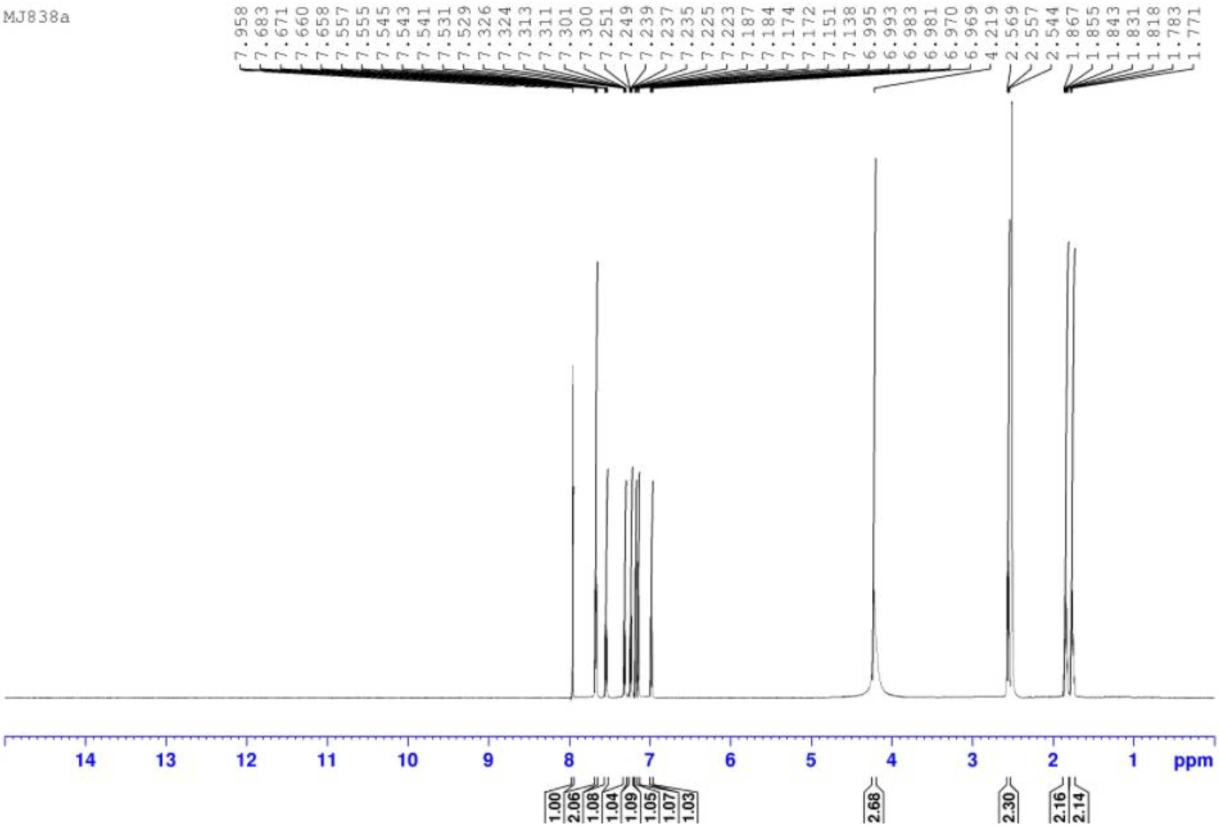
MJ838a
7.958
7.683
7.671
7.660
7.658
7.557
7.555
7.545
7.543
7.541
7.531
7.529
7.326
7.324
7.313
7.311
7.301
7.300
7.251
7.249
7.239
7.237
7.235
7.225
7.223
7.187
7.184
7.174
7.172
7.151
7.138
6.995
6.993
6.983
6.981
6.970
6.969
4.219
2.569
2.557
2.544
1.867
1.855
1.843
1.831
1.818
1.783
1.771
14
13
12
11
10
9
8
7
6
5
4
3
2
1
ppm
1.00
2.06
1.08
1.04
1.09
1.05
1.07
1.03
2.68
2.30
2.16
2.14

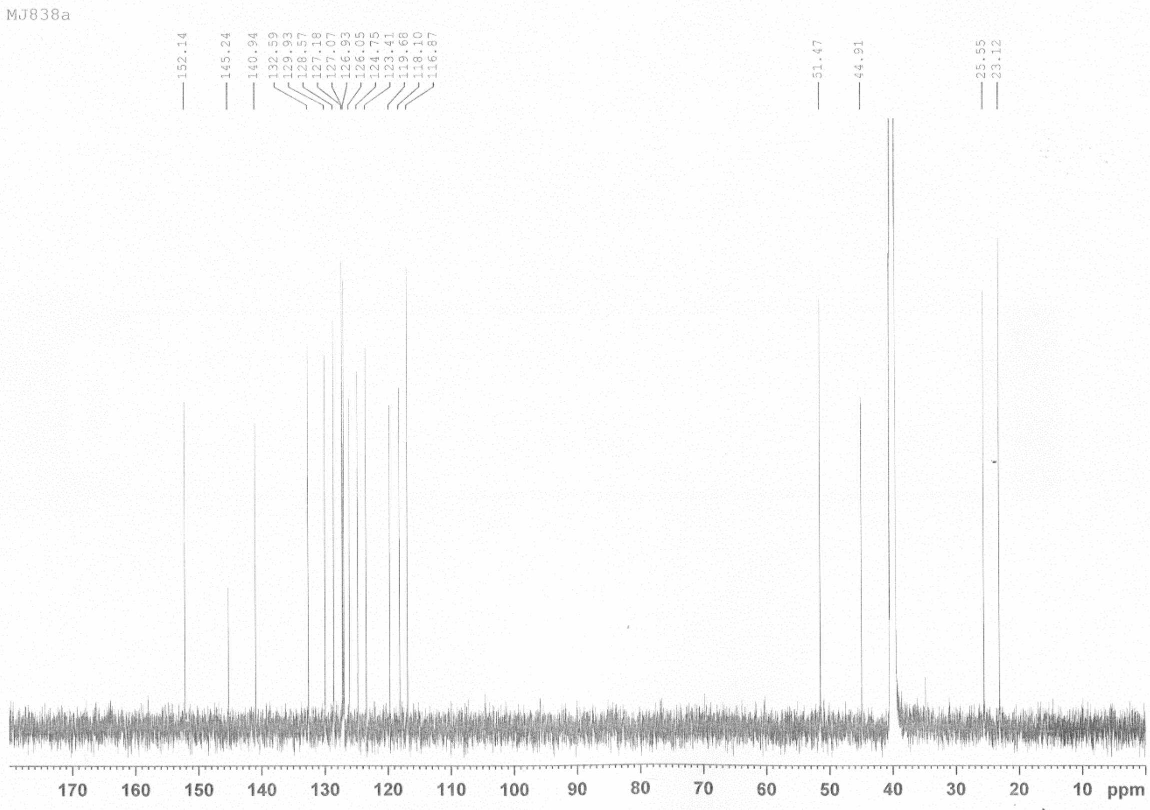

Acquisition Parameter

Source Type	ESI	Ion Polarity	Positive	Set Nebulizer	0.3 Bar
Focus	Active	Set Capillary	4000 V	Set Dry Heater	200 °C
Scan Begin	100 m/z	Set End Plate Offset	-500 V	Set Dry Gas	3.0 l/min
Scan End	1000 m/z	Set Charging Voltage	2000 V	Set Divert Valve	Source
		Set Corona	0 nA	Set APCI Heater	0 °C

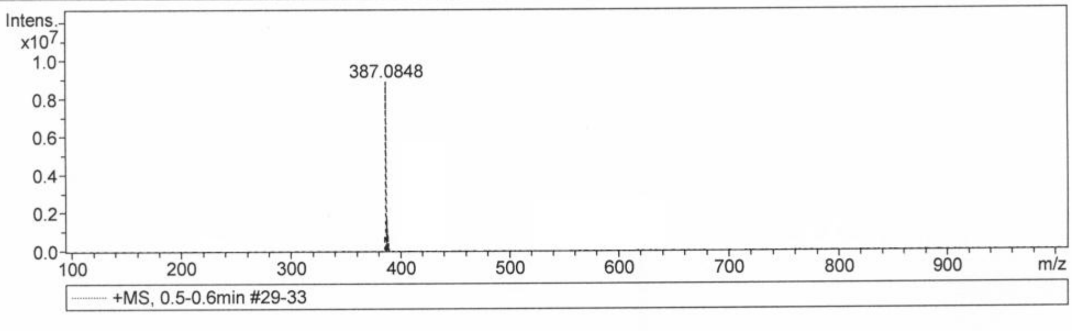

#	m/z	Res.	S/N	I	I %	FWHM
1	387.0848	42101	11833.6	8880034	100.0	0.0092

Compound 10

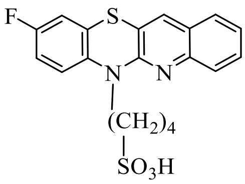
F
S
N
N
(CH2)4
SO3H

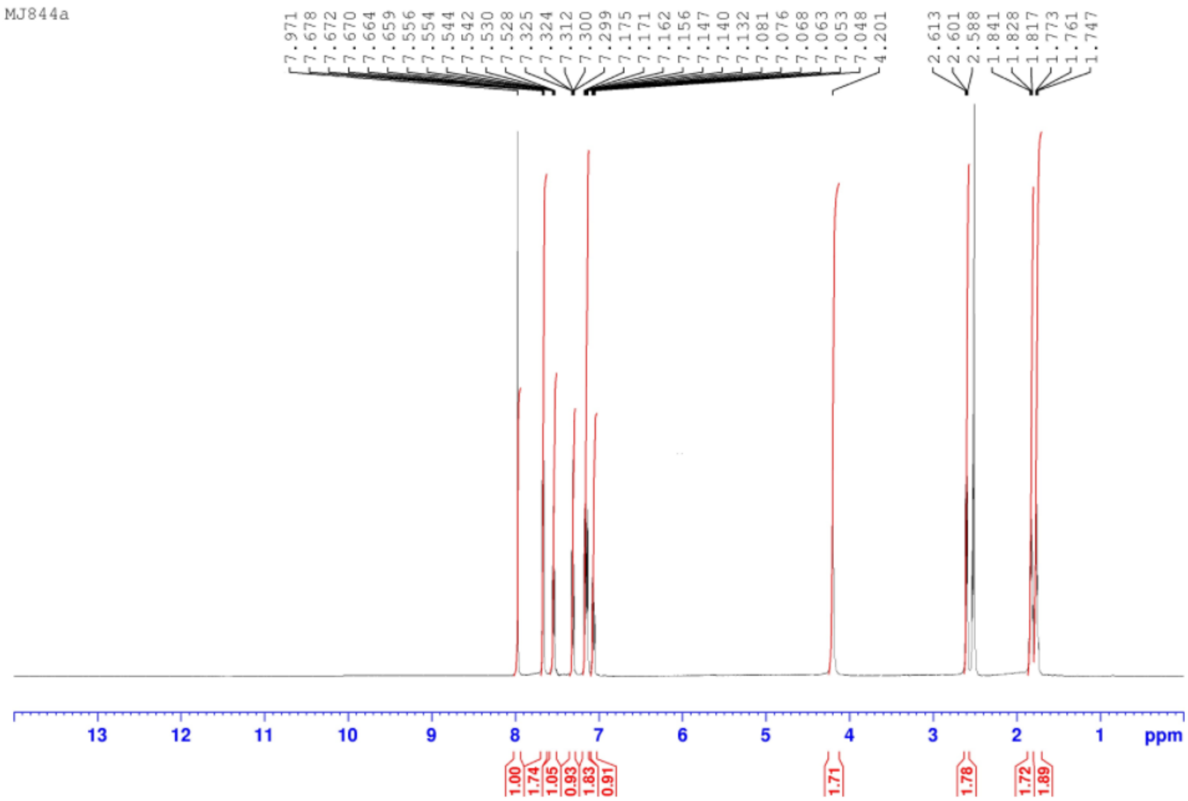
MJ844a
7.971
7.678
7.672
7.670
7.664
7.659
7.556
7.554
7.544
7.542
7.530
7.528
7.325
7.324
7.312
7.300
7.299
7.175
7.171
7.162
7.156
7.147
7.140
7.132
7.081
7.076
7.068
7.063
7.053
7.048
4.201
2.613
2.601
2.588
1.841
1.828
1.817
1.773
1.761
1.747
13
12
11
10
9
8
7
6
5
4
3
2
1
ppm
1.00
1.74
1.05
0.93
1.83
0.91
1.71
1.78
1.72
1.89

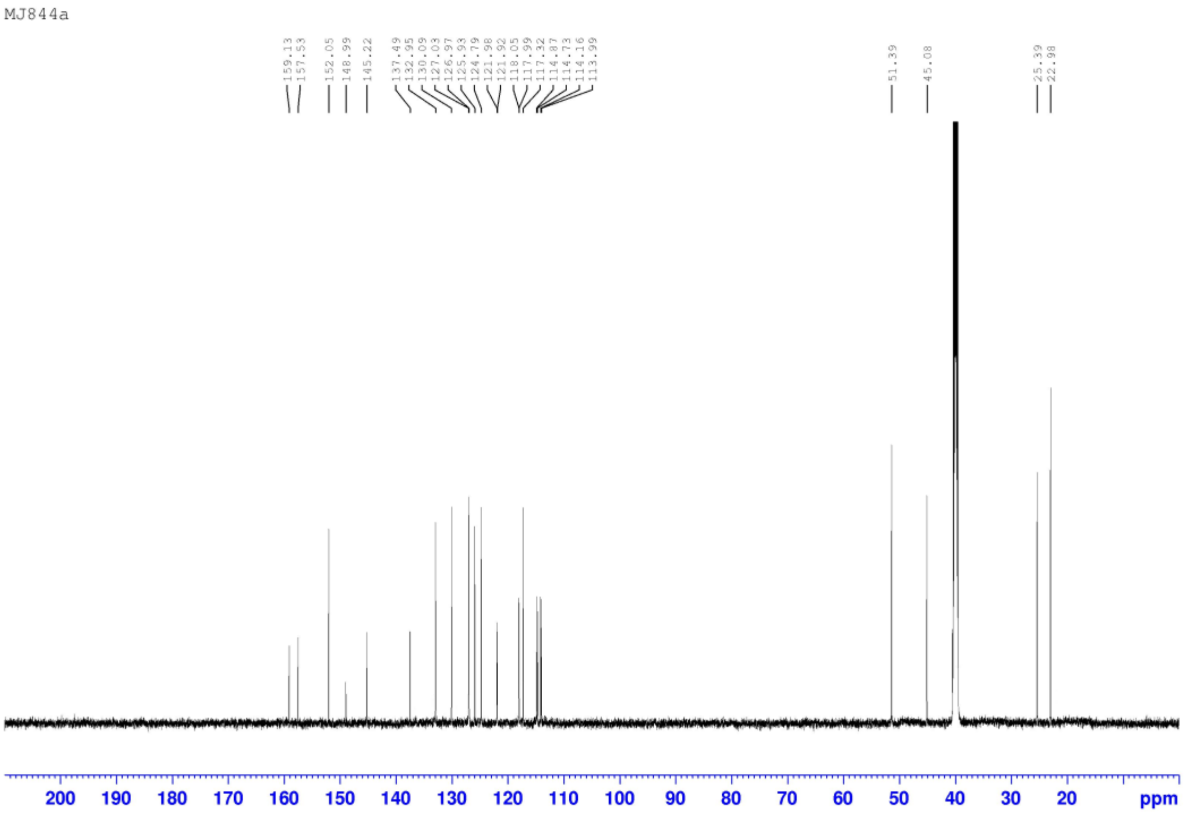

Acquisition Parameter

Source Type	ESI	Ion Polarity	Positive	Set Nebulizer	0.3 Bar
Focus	Active	Set Capillary	4000 V	Set Dry Heater	200 °C
Scan Begin	100 m/z	Set End Plate Offset	-500 V	Set Dry Gas	3.0 l/min
Scan End	1000 m/z	Set Charging Voltage	2000 V	Set Divert Valve	Source
		Set Corona	0 nA	Set APCI Heater	0 °C

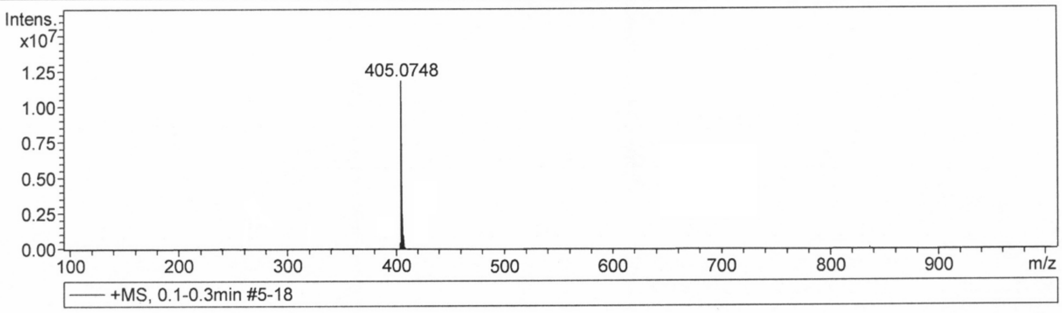

#	m/z	Res.	S/N	I	I %	FWHM
1	405.0748	42905	18643.6	11855851	100.0	0.0094

Compound 11

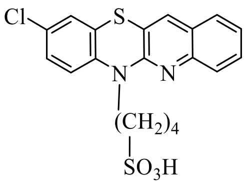
Cl
S
N
N
$(CH_2)_4$
SO_3H

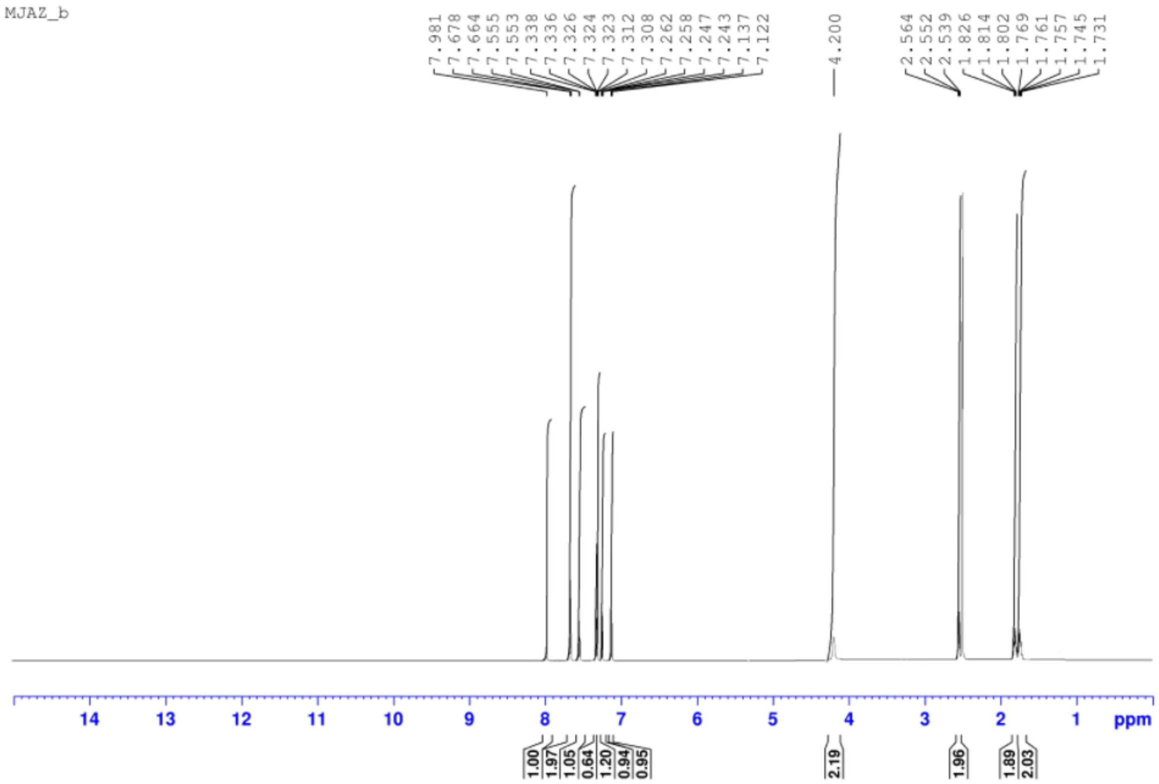
MJAZ_b
7.981
7.678
7.664
7.555
7.553
7.338
7.336
7.326
7.324
7.323
7.312
7.308
7.262
7.258
7.247
7.243
7.137
7.122
4.200
2.564
2.552
2.539
1.826
1.814
1.802
1.769
1.761
1.757
1.745
1.731
14
13
12
11
10
9
8
7
6
5
4
3
2
1
ppm
1.00
1.97
1.05
0.64
1.20
0.94
0.95
2.19
1.96
1.89
2.03

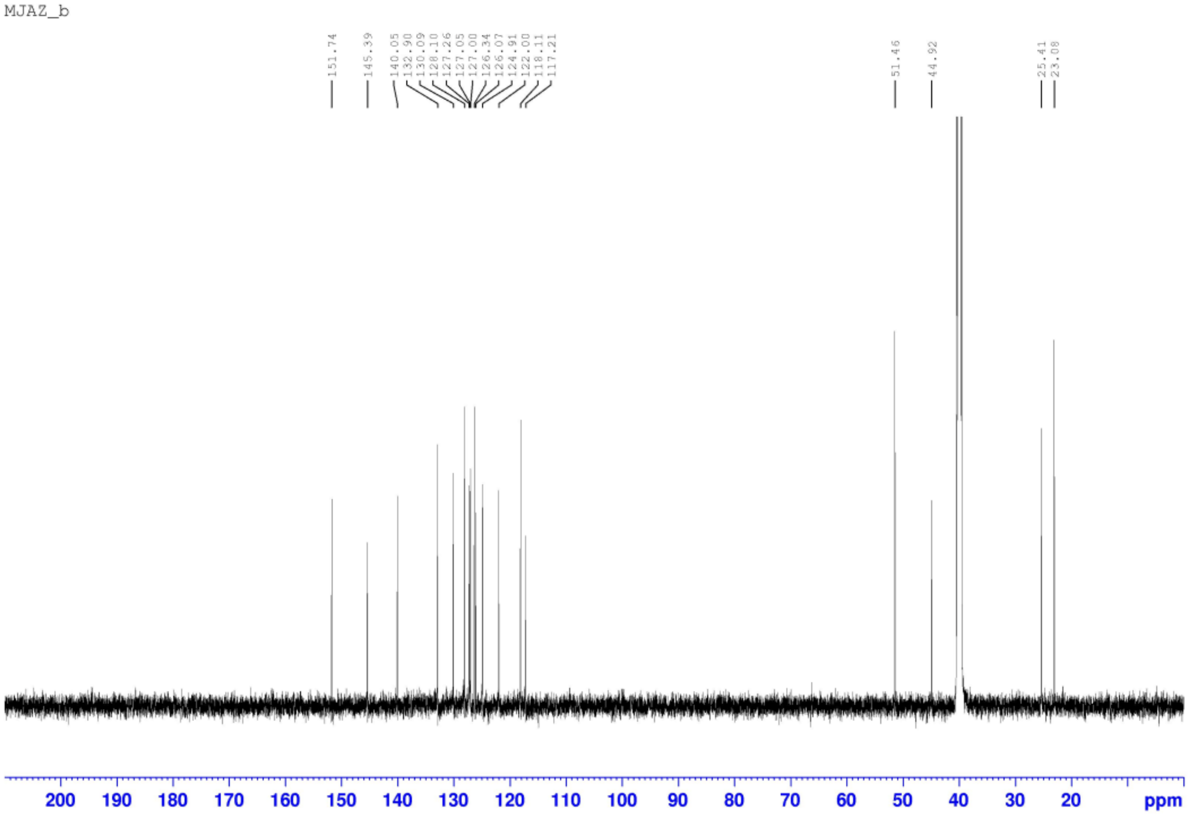

Acquisition Parameter

Source Type	ESI	Ion Polarity	Positive	Set Nebulizer	0.3 Bar
Focus	Active	Set Capillary	4000 V	Set Dry Heater	200 °C
Scan Begin	100 m/z	Set End Plate Offset	-500 V	Set Dry Gas	3.0 l/min
Scan End	1000 m/z	Set Charging Voltage	2000 V	Set Divert Valve	Source
		Set Corona	0 nA	Set APCI Heater	0 °C

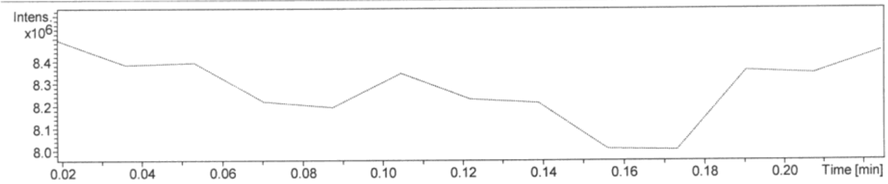

+MS, 0.1-0.2min #7-12

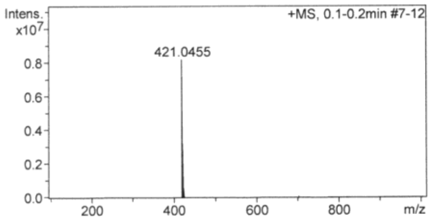

#	m/z	Res.	S/N	I	I %	FWHM
1	421.0455	43319	23885.8	8153817	100.0	0.0097
2	423.0427	39070	9375.8	3215252	39.4	0.0108

Compound 12

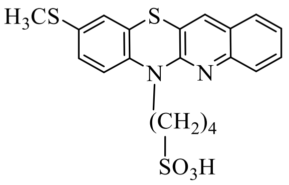
H3CS
S
N
N
(CH2)4
SO3H

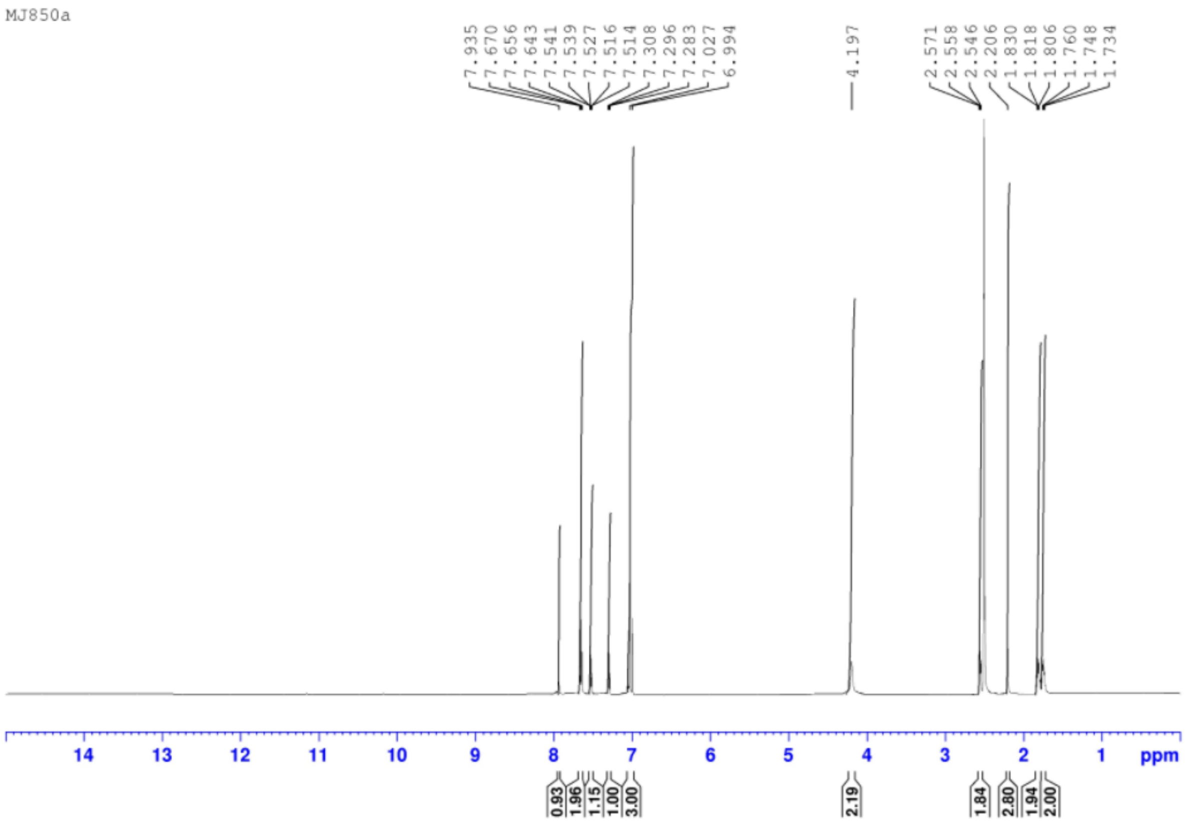
MJ850a
7.935
7.670
7.656
7.643
7.541
7.539
7.527
7.516
7.514
7.308
7.296
7.283
7.027
6.994
4.197
2.571
2.558
2.546
2.206
1.830
1.818
1.806
1.760
1.748
1.734
14
13
12
11
10
9
8
7
6
5
4
3
2
1
ppm
0.93
1.96
1.15
1.00
3.00
2.19
1.84
2.80
1.94
2.00

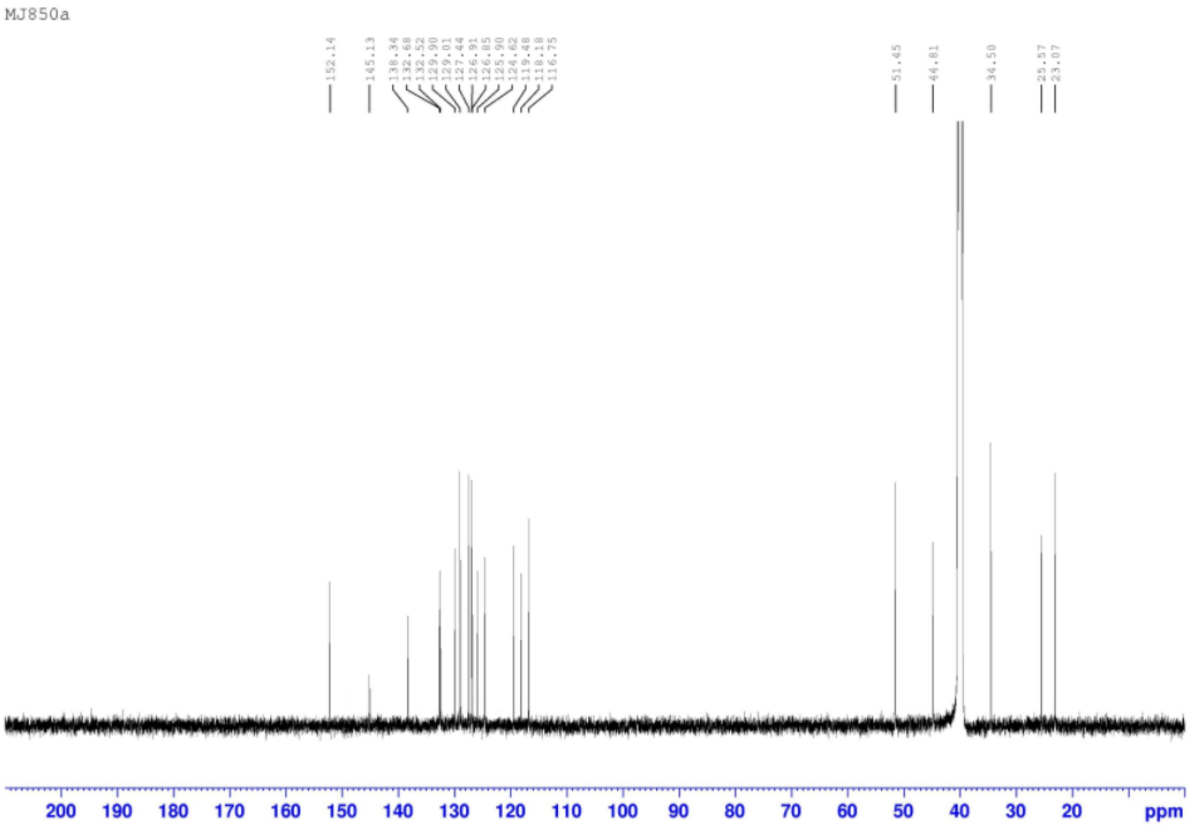

Acquisition Parameter

Source Type	APCI	Ion Polarity	Positive	Set Nebulizer	2.0 Bar
Focus	Active	Set Capillary	4000 V	Set Dry Heater	200 °C
Scan Begin	100 m/z	Set End Plate Offset	-500 V	Set Dry Gas	5.0 l/min
Scan End	3000 m/z	Set Charging Voltage	2000 V	Set Divert Valve	Source
		Set Corona	4000 nA	Set APCI Heater	450 °C

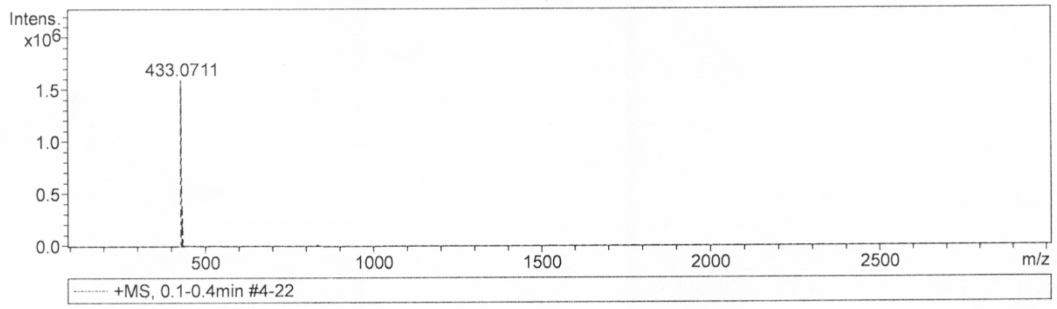

#	m/z	Res.	S/N	I	I %	FWHM
1	433.0711	35375	47233.5	1592535	100.0	0.0122
